# MoSET1 (Histone H3K4 Methyltransferase in *Magnaporthe oryzae*) Regulates Global Gene Expression during Infection-Related Morphogenesis

**DOI:** 10.1371/journal.pgen.1005385

**Published:** 2015-07-31

**Authors:** Kieu Thi Minh Pham, Yoshihiro Inoue, Ba Van Vu, Hanh Hieu Nguyen, Toru Nakayashiki, Ken-ichi Ikeda, Hitoshi Nakayashiki

**Affiliations:** Laboratory of Cell Function and Structure, Graduate School of Agricultural Science, Kobe University, Nada Kobe, Japan; Oregon State University, UNITED STATES

## Abstract

Here we report the genetic analyses of histone lysine methyltransferase (KMT) genes in the phytopathogenic fungus *Magnaporthe oryzae*. Eight putative *M*. *oryzae* KMT genes were targeted for gene disruption by homologous recombination. Phenotypic assays revealed that the eight KMTs were involved in various infection processes at varying degrees. *Moset1* disruptants (*Δmoset1*) impaired in histone H3 lysine 4 methylation (H3K4me) showed the most severe defects in infection-related morphogenesis, including conidiation and appressorium formation. Consequently, *Δmoset1* lost pathogenicity on wheat host plants, thus indicating that H3K4me is an important epigenetic mark for infection-related gene expression in *M*. *oryzae*. Interestingly, appressorium formation was greatly restored in the *Δmoset1* mutants by exogenous addition of cAMP or of the cutin monomer, 16-hydroxypalmitic acid. The *Δmoset1* mutants were still infectious on the super-susceptible barley cultivar Nigrate. These results suggested that MoSET1 plays roles in various aspects of infection, including signal perception and overcoming host-specific resistance. However, since *Δmoset1* was also impaired in vegetative growth, the impact of MoSET1 on gene regulation was not infection specific. ChIP-seq analysis of H3K4 di- and tri-methylation (H3K4me2/me3) and MoSET1 protein during infection-related morphogenesis, together with RNA-seq analysis of the *Δmoset1* mutant, led to the following conclusions: 1) Approximately 5% of *M*. *oryzae* genes showed significant changes in H3K4-me2 or -me3 abundance during infection-related morphogenesis. 2) In general, H3K4-me2 and -me3 abundance was positively associated with active transcription. 3) Lack of MoSET1 methyltransferase, however, resulted in up-regulation of a significant portion of the *M*. *oryzae* genes in the vegetative mycelia (1,491 genes), and during infection-related morphogenesis (1,385 genes), indicating that MoSET1 has a role in gene repression either directly or more likely indirectly. 4) Among the 4,077 differentially expressed genes (DEGs) between mycelia and germination tubes, 1,201 and 882 genes were up- and down-regulated, respectively, in a *Moset1*-dependent manner. 5) The *Moset1*-dependent DEGs were enriched in several gene categories such as signal transduction, transport, RNA processing, and translation.

## Author Summary

This paper provides two major contributions to the field of genetics. First, we systematically studied the biological roles of eight histone lysine methyltransferase (KMT) genes in the phytopathogenic fungus *Magnaporthe oryzae*. We investigated their roles, especially focusing on their involvement in infection-related morphogenesis and pathogenicity. The results showed that the eight KMTs were involved in various infection processes to varying degrees, and that MoSET1, one of the KMTs catalyzing methylation at histone H3 lysine 4 (H3K4), had the largest impact on the pathogenicity of the fungus. Second, we focused on the role of MoSET1 in global gene regulation. H3K4 methylation is generally believed to be an epigenetic mark for gene activation in higher eukaryotes. However, in *Saccharomyces cerevisiae*, SET1 was originally characterized as being required for transcriptional silencing of silent mating-type loci. We addressed this apparent discrepancy by examining genome-wide gene expression and H3K4 methylation during infection-related morphogenesis in *M*. *oryzae*. RNA-seq analysis of a MoSET1 deletion mutant revealed that MoSET1 was indeed required for proper gene activation and repression. ChIP-seq analyses of H3K4 methylation and MoSET1 suggested that MoSET1 could directly play a role in gene activation while MoSET1-dependent gene repression may be caused by indirect effects.

## Introduction

In eukaryotic cells, DNA-dependent processes can be regulated by covalent modifications of histones such as methylation, acetylation, phosphorylation, sumoylation, and ubiquitination [1]. Long amino-terminal tails of histones protruding from nucleosome cores are especially subject to post-translational modifications. The combination of histone modifications to regulate cellular processes is a dynamic language, and is referred to as the histone code [1]. Histone modifications serve as marks for the recruitment of various chromatin proteins or protein complexes to modulate diverse chromatin functions including gene expression, silencing, repair, and replication [2]. Numerous “writing” enzymes (methylases, acetylases etc) and “erasing” enzymes (demethylases, deacetylases etc) are involved in the histone code.

Histone methyltransferases are a group of enzymes catalyzing the transfer of methyl groups from *S*-adenosyl methionine to histones. They can be divided into two groups based on their target amino acid residues: protein arginine methyltransferases (RMTs) and histone lysine methyltransferases (KMTs) [3–5]. A nomenclature system for the KMT family has recently been proposed, in which KMTs are classified into eight major subclasses, KMT1 to KMT8, based on their phylogenetic relationships and domain structure/organization [6]. For example, KMT1 proteins, exemplified by *Drosophila melanogaster* Su(Var)3-9, *Schizosaccharomyces pombe* Clr4, and *Neurospora crassa* DIM-5, specifically methylate H3K9, which leads to gene silencing and heterochromatin formation [7–9]. The KMT2 proteins, typified by *Saccharomyces cerevisiae* SET1 and *D*. *melanogaster* Trithorax, specifically catalyze methylation at H3K4, a mark for gene activation [10, 11]. Since all KMTs except the KMT4 class contain a SET domain, named after three *Drosophila* lysine methyltransferases: Su(var)3-9, Enhancer of zeste, and Trithorax, they are also often referred to as SET proteins. It is to be noted that there are also known possible KMT proteins that are not included in the nomenclature system such as SET3 and SET4 in *S*. *cerevisiae*.

KMTs are conserved in a wide range of eukaryotes, playing roles in cellular signaling pathways related to the cell cycle, cell motility, transcription, apoptosis, and cancer [12, 13]. In filamentous fungi, KMT-related gene regulation has been investigated mainly with regard to gene silencing and secondary metabolite (SM) production [9, 14–21]. In *N*. *crassa*, H3K9me3 catalyzed by DIM-5 belonging to the KMT1 class directs DNA methylation and heterochromatin formation by recruiting a protein complex containing heterochromatin protein-1 (HP1) and DIM-2 DNA methyltransferase through interaction of the chromo shadow domain of HP1 and PXVXL-like motifs in DIM-2 [9]. In *Fusarium graminearum*, H3K27me3 catalyzed by KMT6 was required for normal fungal development and contributed to regulating the “cryptic genome” including SM gene clusters [18]. Gene repression by H3K9 and H3K27 methylation was also recently shown to be involved in fungal symbiosis and pathogenicity through production of SM and effectors [19, 20].

In *Aspergillus nidulans*, H3K4me2 and H3K4me3, marks for gene activation play a role in chromatin-level regulation of SM gene clusters [21]. A loss-of-function mutation of the CclA gene, a member of the H3K4 methylating COMPASS (Complex Proteins Associated with Set1), resulted in a reduction of H3K4me2 and H3K4me3 at the SM gene clusters [22]. Surprisingly, cryptic SM gene clusters are activated in the *ΔcclA* mutant despite H3K4me2 and H3K4me3 being considered marks for gene activation [22]. While it is generally believed that H3K4 di- and tri-methylation are epigenetic marks for gene activation in higher eukaryotes, involvement of H3K4 methylation in gene repression is also reported in fungi and other organisms [21–25]. To date, it is not clearly known to what extent genes are up- or down-regulated in a H3K4 methylation-dependent manner, and what is the underlying mechanism for this apparent discrepancy.

Rice blast caused by *Magnaporthe oryzae* (*Pyricularia oryzae*) is one of the most devastating worldwide rice (*Oryza* spp.) diseases. This fungal species consists of several host-specific pathotypes that cause blast disease on a wide range of gramineous hosts including wheat, oat, finger millet and etc. Owing to their economic importance and genetic tractability, rice and *M*. *oryzae* have emerged as a model system for studying fungi-plant interactions [26]. *M*. *oryzae* displays dramatic morphological changes during infection [27]. When a fungal spore lands on a plant’s surface it germinates and forms a melanized dome-shaped infection structure, called an appressorium, at the tip of the germ tube. The appressorium generates enormous turgor pressure and physical force to breach the host cuticle, and the fungus eventually develops invasive hyphae to colonize host cells. These morphological changes are accompanied with global transcriptional alterations [28–30]. However, the involvement of genome-wide histone modifications in infection-related transcriptional alterations is poorly understood in *M*. *oryzae*. We recently demonstrated that MoSET1 catalyzing H3K4 methylation was required for substrate-induced transcriptional activation of the MoCel7C cellulase gene in *M*. *oryzae* [31]. Here we report a reverse genetics study of the KMT gene family in the *M*. *oryzae* genome, and examine their roles in global gene regulation related to the formation of infection structures in *M*. *oryzae*, especially focusing on that of MoSET1.

## Results

### Generation of *M*. *oryzae* deletion mutants of KMT genes using the split-marker gene deletion method

Eight putative KMT genes were identified in the *M*. *oryzae* genome based on sequence similarity and domain structure of known KMTs in the KEGG database (http://www.genome.ad.jp/kegg). *Moset1* (MGG_15053) belonging to the KMT2 family, was previously named after *S*. *cerevisiae Set1* [31]. The other seven genes were designated in this study as *Mokmt1* (MGG_06852), *Mokmt3* (MGG_01661), *Mokmt4* (MGG_05254), *Mokmt5* (MGG_07393), *Mokmt6* (MGG_00152), *Mokmt2h* (MGG_02937), and *Moset6* (MGG_15522) (Table 1). *Mokmt1*, *Mokmt3*, *Mokmt4*, *Mokmt5*, and *Mokmt6* likely belong to corresponding KMT families [6]. MoKMT2H and MoSET6 showed amino acid sequence similarity with *N*. *crassa* SET-3 and *Schizosaccharomyces pombe* SET6, respectively.

**Table 1:** Putative *M*. *oryzae* histone lysine methyltranferases (KMTs) used in this study.

Gene ID	Protein Name	Protein motif	Homolog	function
MGG_06852	MoKMT1	Pre-SET motif and SET domain	*N*.*crassa* DIM-5	H3K9me
MGG_15053	MoSET1	RNA recognition motif and SET domain	*S*.*cerevisiae* SET1	H3K4me
MGG_01661	MoKMT3	SET domain	*N*.*crassa* SET-5	
MGG_05254	MoKMT4	DOT1-like	*S*.*cerevisiae* DOT1	
MGG_07393	MoKMT5	SET domain	*S*. *pombe* Set9	H4K20me
MGG_00152	MoKMT6	SET domain	*D*. *melanogaster* EZH2	H3K27me
MGG_02937	MoKMT2H	SET domain	*N*.*crassa* SET-3	
MGG_15522	MoSET6	SET domain	*S*. *pombe* Set6	

To determine biological functions of *M*. *oryzae* KMT genes, deletion mutants were constructed using the split-marker recombination method [32] (S1–S7 Figs). Ectopic transformants, which had an insertion of a disruption construct somewhere in the genome other than the target locus, and gene complementation strains, which were deletion mutant-derived strains complemented by random insertion of a plasmid carrying the corresponding wild-type locus, were also created. These strains were used in further studies alongside the deletion mutants (S1–S7 Figs and S1 Table). Deletion mutants and a complementation strain of *Moset1* were previously made and used in this study [31].

**Fig 1:**
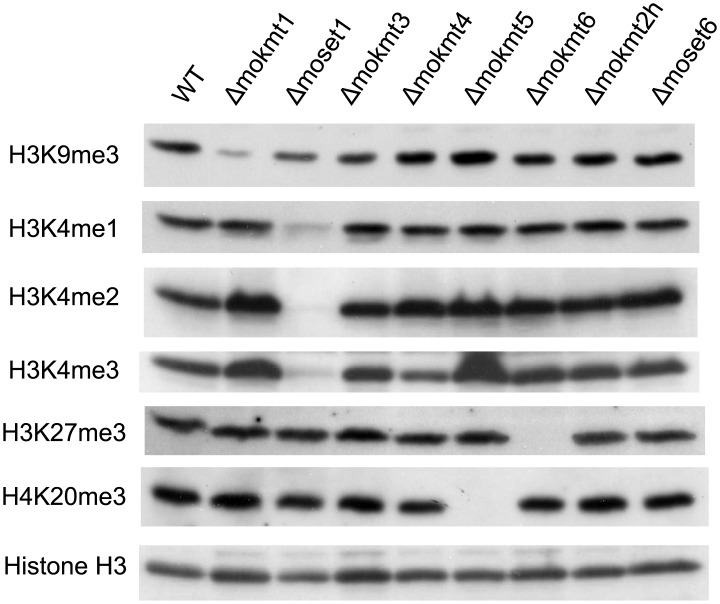
Western blotting analysis of histone modifications in KMT mutants. Total protein extracted from *M*. *oryzae* cells was subjected to 15% SDS polyacrylamide gel electrophoresis, and probed with antibodies against H3K4me1 (Active Motif #39298), H3K4me2 (Active Motif #39141), H3K4me3 (Active Motif #39159), H3K9me3 (Active Motif #39161), H4K20me3 (Active Motif #39181), and a C-terminal peptide of histone H3 (Active Motif #39163).

Histone lysine methylation levels in the KMT deletion mutants were assessed using western blotting with specific antibodies (Fig 1). In the *Δmoset1* mutant, the levels of H3K4me2 and H3K4me3 were strongly reduced, while H3K4me1 moderately decreased. Abundance of signals for H3K9me3, H3K27me3 and H4K20me3 was also significantly reduced in the*Δmokmt1*, *Δmokmt6*, and *Δmokmt5* mutants, respectively. These target sites for *Magnaporthe* KMTs were consistent with known target sites for corresponding KMT family proteins in other organisms. Western blotting was also used to test antibodies against H3K14me2 (active motif #39350), H3K36me3 (active motif #61102), and H3K79me2 (active motif #39144), however, no specific signal reduction in any KMT deletion mutant was detected (S8 Fig). H3K36 and H3K79 methylation are known to be catalyzed by Set2 (KMT3 family) and Dot1 (KMT4 family), respectively, in *S*. *cerevisiae*. Other KMT proteins might be involved in these histone marks in *M*. *oryzae*, or the specificity of the antibodies might not be strict enough to distinguish between marked and non-marked histones in *M*. *oryzae*.

The decreased levels of histone methylation in the deletion mutants were completely recovered in gene complementation strains (S9 Fig). These results indicated that MoKMT1, MoSET1, MoKMT5, and MoKMT6 catalyze methylation of H3K9, H3K4, H4K20, and H3K27 respectively, in *M*. *oryzae*.

### *M*. *oryzae* KMT deletion mutants showed defects in infection-related morphogenesis and pathogenicity to host plants at varying degrees

**Fig 2:**
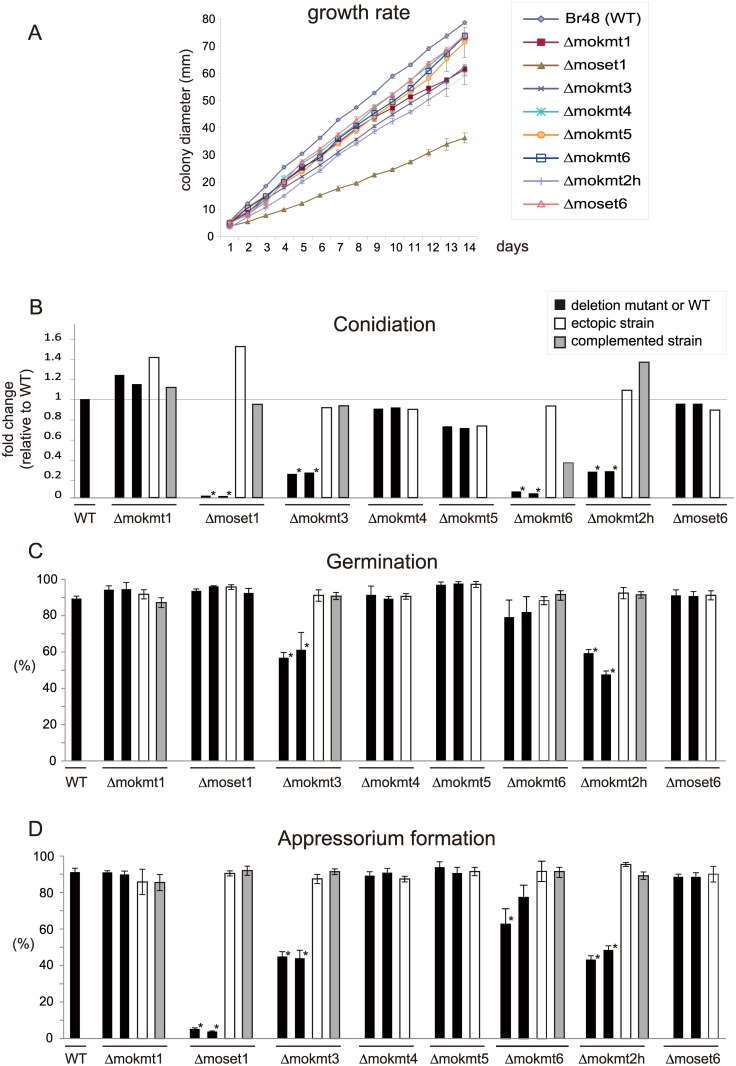
Phenotypic characterization of KMT mutants of *Magnaporthe oryzae*. (A) Vegetative growth was measured for 14 days on complete agar medium. (B) Conidiation was measured by counting the number of conidia harvested 3 days after BLB induction by suspending them with 20 ml of sterile distilled water per plate. (C) The rate of conidial germination on hydrophobic surfaces was measured under a light microscope. (D) Appressorium formation was measured as the percentage ratio of appressorium-forming mycelium to germinating mycelium on hydrophobic surfaces after 24 h incubation at 25°C. (B-D) Black bars indicate the wild-type strain Br48 (WT) or the original deletion mutants. White bars represent ectopic strains that have an insertion of a disruption construct somewhere in the genome other than the target locus. Grey bars indicate complemented strains that have random insertion of a plasmid carrying the corresponding wild-type locus in a deletion mutant background. All data are presented as means ± SD from three independent experiments. The order of the *M*. *oryzae* strains appeared in the figure is the same as that given in S1 Table. ND, not determined; *, significant difference (*p*< 0.01, two-tailed t-test after angular transformation).

The rates of conidiation, germination, and appressorium formation were assessed for phenotypical characterization of the KMT mutants with regards to infection. In addition, their growth rates were examined on rich media. The assay used two deletion mutants and one ectopic transformant for each KMT gene, with a complement strain employed when phenotypic defects were observed. The growth rates of the KMT-mutants were generally lower than that of the wild-type strain (Fig 2A). Especially, *Δmoset1* exhibited the most severe reduction in vegetative growth, conidiation and appressorium formation but not in germination (Fig 2B–2D). The*Δmokmt3*, and*Δmokmt2h* mutants showed moderate defects in all phenotypic traits investigated in Fig 2. TheΔmokmt6 mutants also showed severe reduction in conidiation and slight defects in appressorium formation (Fig 2B and 2D). Compared with the wild-type strain, the rates of conidiation and appressorium formation were reduced to less than 10% in the *Δmoset1* mutants, and to 20–50% in the*Δmokmt3* and*Δmokmt2h* mutants (Fig 2B–2D). The rate of germination was decreased by 40–50% in the*Δmokmt3* and*Δmokmt2h* mutants. Interestingly, while the *Δmoset1* mutants germinated at levels comparable to the wild-type, the conidia of *Δmoset1* mutants often appeared to be malformed. The wild-type strain produced three-celled, tear-drop-shaped spores. Conidia of the *Δmoset1* mutants were also three-celled, but were more elongated than the wild-type spores. All phenotypic defects observed in the*Δmoset1*, *Δmokmt3*, *Δmokmt6*, and*Δmokmt2h* mutants recovered to wild-type levels in the corresponding complement strains with an exception (conidiation in*Δmokmt6*), indicating that the KMT mutant phenotypes were caused by the corresponding KMT genes.

Infection assays of the KMT mutants were performed using three wheat and two barley cultivars with different levels of resistance/susceptibility to the wild-type wheat-infecting *M*. *oryzae* strain (Br48) used in this study. The order of susceptibility of the cultivars to Br48 was as follows: barley, Nigrate (super susceptible) > barley, Russian No. 74 ≈ wheat, Norin 4 (susceptible) > wheat, Chinese spring (moderate susceptible) > wheat, Thatcher (moderate resistant) [33].

**Fig 3:**
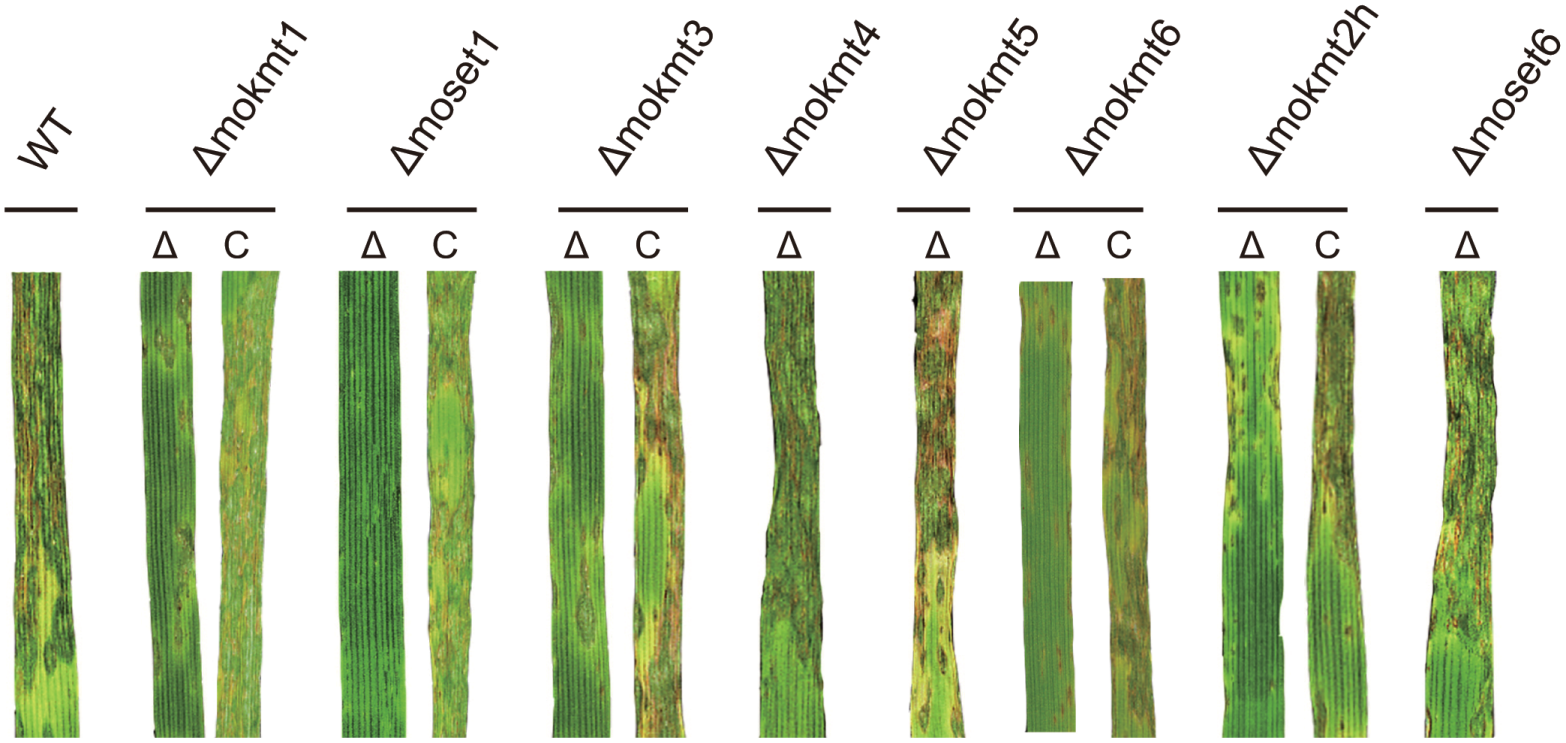
Inoculation test of KMT deletion mutants and their complemented strains. Infection assay was performed using the wheat cultivar Norin 4 at 22°C. Five days after inoculation, symptoms on the inoculated plants were evaluated by a grading method (see Materials & Methods). This experiment was repeated at least three times, and representative samples are presented.

Consistent with the rates of appressorium formation, pathogenicity to the wheat and barley cultivars was most severely impaired in the *Δmoset1* mutants (Fig 3 and Table 2). The *Δmoset1* mutants produced no visible symptoms on most host plants tested. Interestingly, the *Δmoset1* mutants caused disease, albeit with fewer lesions, on the super susceptible barley cultivar Nigrate (S10 Fig), indicating that mutants did not completely lose their ability to infect plants. That fewer lesions were produced by the *Δmoset1* mutants could largely be attributed to the low rates of appressorium formation.

**Table 2:** Infection types in wheat and barley cultivars inoculated with the wild type and mokmt deletion strains.

	Infection type (22°C)				
Fungal strain	Wheat cultivar			Barley cultivar	
	Thatcher	Chinese spring	Norin 4	Nakaizumi-zairai	Nigrate
Br48	2B	4BG	4BG	4BG	5G
*Δmokmt1*.9	1B	2B	3BG	3BG	5G
*Δmokmt1*.13	1B	3BG	3BG	3BG	5G
MoKMT1E	2B	4BG	4BG	4BG	5G
*Δmoset1*.35	0	0	0	0	3BG
*Δmoset1*.36	0	0	0	0	3BG
MoSET1E	3B	4BG	4BG	4BG	5G
*Δmokmt3*.22	2B	2B	3B	2BG	5G
*Δmokmt3*.26	1B	2B	3BG	3BG	5G
MoKMT3E	2B	3BG	4BG	4BG	5G
*Δmokmt4*.17	2B	3BG	4BG	4BG	5G
*Δmokmt4*.19	2B	3BG	4BG	4BG	5G
MoKMT4E	2B	3BG	4BG	4BG	5G
*Δmokmt5*.2	2B	4BG	4BG	4BG	5G
*Δmokmt5*.4	2B	4BG	4BG	4BG	5G
MoKMT5E	2B	4BG	4BG	4BG	5G
*Δmokmt6*.5	N.D.	2B	3Bg	N.D.	5G
*Δmokmt6*.10	N.D.	3Bg	4bG	N.D.	5G
MoKMT6E	2B	4bg	4bG	4bG	5G
*Δmokmt2h*.8	1B	1B	2BG	2B	5G
*Δmokmt2h*.13	1B	1B	2BG	2B	5G
MoKMT2hE	2B	4BG	4BG	4BG	5G
*Δmoset6*.14	2B	4BG	4BG	4BG	5G
*Δmoset6*.16	2B	3BG	4BG	4BG	5G
MoSET6E	2B	3BG	4BG	4BG	5G
MoKMT1C	2B	4BG	4BG	4BG	5G
MoSET1C	2B	3BG	4BG	4BG	5G
MoKMT3C	2B	3BG	4BG	4BG	5G
MoKMT6C	N.D.	3BG	4BG	N.D.	5G
MoKMT2hC	3B	4BG	4BG	4BG	5G

- The size of lesion: 0, no visible evidence of infection; 1, pinpoint spots; 2, small lesion (<1.5 mm); 3, lesion with an intermediate size (<3 mm); 4, large and typical lesion; 5, complete blighting of leaf blades.

- The color of lesion: G: green, B: brown. 0 to 3B: resistant; 3G to 5G: susceptible

*Δmokmt1*, *Δmokmt3*, *Δmokmt6* and*Δmokmt2h* mutants showed significant reduction in pathogenicity on all tested plant cultivars except Nigrate (Table 2). *Δmokmt2h* mutants failed to cause compatible lesions on any plants other than Nigrate, indicating that mutants became non-pathogenic with certain host plants susceptible to the parent strain Br48. *Δmokmt3* mutants also become non-pathogenic with the wheat cultivar Chinese spring, which is moderately susceptible to Br48. The other strains, including the*Δmokmt4*, *Δmokmt5* and*Δmoset6* mutants, ectopic transformants, and complement strains were infectious to all plant cultivars at levels comparable to the wild-type strain.

To further examine infection types of the KMT mutants, cytological analysis of inoculated leaves of the wheat cultivar Norin 4 was performed. At least 100 spores with an appressorium were assessed, and cytological interactions classified into four types: A, B, C, and D [34]. In Type A, no reaction of the host cells was observed. In Type B, inhibition of fungal growth was associated with papilla, a cell wall apposition at the penetration site. Type C represents the hypersensitive reaction of epidermal cells. Types A to C are resistance responses of host cells. Type D describes a susceptible response where infection hyphae were observed in infected cells.

In leaves infected with the wild type strain, the incidence of susceptible response Type D was predominant (63.9%) (Table 3). Similarly, Type D was predominant in leaves infected with the*Δmokmt4*, *Δmokmt5*, and*Δmoset6* mutants at levels similar to the wild-type strain. In contrast, the rate of resistant responses (Types A + B + C) was the majority in leaves infected with the*Δmokmt1*, *Δmoset1*, *Δmokmt3*, *Δmokmt6* and*Δmokmt2h* mutants (Table 3). In leaves inoculated with the*Δmoset1* mutant, Type A (no reaction) was predominant, (77.3%), suggesting that this mutant mostly failed to penetrate plant cuticle and/or cell walls. The*Δmokmt3* and*Δmokmt2h* mutants induced cytological responses at very similar rates in the host cells. Infection by the two mutants was mostly prevented by the HR (~60%), and partly blocked at the papilla (~20%). Only a small percentage of germlings successfully formed invasive hyphae in infected cells (Table 3). The*Δmokmt1* and*Δmokmt6* mutant showed a slight reduction in compatible interaction rate (Type D), and slight increases in incompatible interaction rates (Type A–C) (Table 3).

**Table 3:** Cellular response of wheat leaves cultivar Norin 4 to the wild type and mokmt deletion strains.

	Percentage of cytological reaction (%)				HR ratio
Fungal strains	No reaction (A)	Papilla formation (B)	HR (C)	Hyphal growth (D)	(C/C+D) (%)
Br48	3.6	5.8	26.7	63.9	29.5
*Δmokmt1*.9	9.9	6.9	34.7	48.5	41.7
*Δmoset1*.36	77.3	21.3	1.4	0	100
*Δmokmt3*.22	20.7	18.0	55.3	5.8	90.5
*Δmokmt4*.19	5.3	5.2	25.3	61.1	29.3
*Δmokmt5*.4	3.2	4.9	26	67.3	27.9
*Δmokmt6*.5	7.9	17.6	34.3	40.2	46.1
*Δmokmt2h*.13	16.7	19.2	60.3	3.8	94.1
*Δmoset6*.14	7.1	6.4	28.1	59.4	32.6

HR; hypersensitive response

The order of the degrees of reduction in KMT-mutant pathogenicity was as follows: *Δmoset1* >*Δmokmt2h* >*Δmokmt3* >*Δmokmt1≈Δmokmt6*. The KMT mutants*Δmokmt4*, *Δmokmt5*, and*Δmoset6* showed no detectable differences from the wild-type strain in all infection assays in this study. Therefore, we concluded that MoSET1 played the most important role in infection-related morphogenesis in *M*. *oryzae*, and focused on MoSET1 for our further studies.

### Exogenous signal molecules restored the defects of infection-related morphogenesis in *Δmoset1* mutants but not its pathogenicity to the host plant

**Fig 4:**
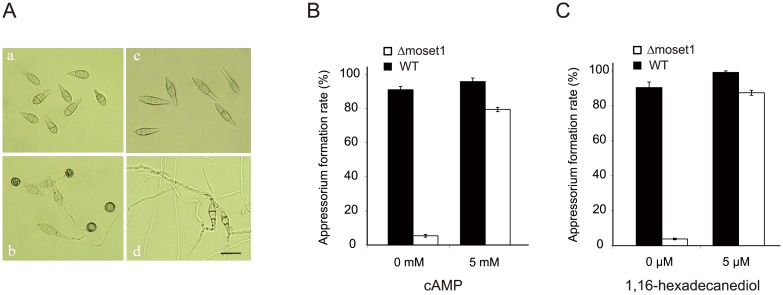
The defect of *Δmoset1* mutants in appressorium formation was restored by exogenous signal molecules. (A) Effect of *Moset1* deletion on conidial morphology and appressorium formation. Conidia (a) and appressoria (b) of *M*. *oryzae* wild-type strain Br48. Conidia of the *Δmoset1* mutant showed an elongated shape (c) and seldom formed an appressorium (d). Bar = 10 μm. Exogenous addition of 5mM cAMP (B) and 5μM 1,16-hexadecanediol (C) restored appressorium formation in the *Δmoset1* mutant. Error bars represent standard deviation.

Pharmacological examination was performed to gain an insight into which stage in the signaling pathway leading to appressorium formation was blocked in *Δmoset1* mutants. Chemical and physical signals from host plants can trigger infection-related morphogenesis in *M*. *oryzae*. One such chemical signal is 1, 16-hexadecanediol, a plant cutin monomer released from the plant cuticle by degradation enzymes produced by the fungus [35]. After perception of external signals, the secondary messenger cyclic AMP (cAMP) plays a crucial role in the signaling pathway leading to appressorium formation in *M*. *oryzae* [36, 37]. Therefore, the effects of 1, 16-hexadecanediol and cAMP on infection-related morphogenesis in the *Δmoset1* mutant were examined. In the presence of 5 mM cAMP or 5 μM 1, 16-hexadecanediol, the rates of appressorium formation were greatly restored (to over 80%) in the *Δmoset1* mutant, though rates were still lower than seen in the wild-type strain (Fig 4B and 4C). It is noteworthy that there was no significant difference in the rate of appressorium formation between treatments with cAMP and 1, 16-hexadecanediol, suggesting that *Δmoset1* mutants may have defects in the production and/or perception of external signals from host plants.

Inoculation assays were performed to determine whether the addition of exogenous 1, 16-hexadecanediol and cAMP restored the pathogenicity of *Δmoset1* mutants. 5 mM cAMP or 5 μM 1, 16-hexadecanediol was added to conidia suspension of *Δmoset1* mutants, and then spotted on leaves of the susceptible wheat cultivar Norin 4. No symptoms were observed on leaves inoculated with the chemical treatments (S11A Fig), suggesting that defects in appressorium formation were not the only cause making the *Δmoset1* mutant noninfectious in wheat. To further examine this finding, wound inoculation tests were performed on Norin 4. The *Δmoset1* mutant failed to cause disease on the wounded leaves (S11B Fig), suggesting that the *Δmoset1* mutant had some deficits in its ability to develop disease, even after entering into plant tissues. Overall, these results suggested that MoSET1 is involved in the regulation of genes required for external signaling perception and disease development in plant cells.

### Methylation of H3K4 is associated with transcriptionally active genes

Chromatin immunoprecipitation sequencing (ChIP-seq) and RNA sequencing (RNA-seq) analyses [38], using chromatin and RNA samples extracted from vegetative mycelia and germination tubes of the wild-type and*Δmoset1* strains, were performed to examine genome-wide H3K4 methylation and MoSET1 distribution patterns during infection-related morphogenesis, and to determine their relationships with gene expression (S2 Table). Germination tubes were collected after 6 h incubation, when appressoria had begun to form; genes involved in appressorium formation were expected to be at their most active at this time point. To carry out ChIP-analysis of MoSET1, N-terminal FLAG-tagged MoSET1 was constructed and introduced into the*Δmoset1* mutant. The phenotypic defects of theΔmoset1 mutant recovered when FLAG-tagged MoSET1 was introduced in the mutant (S12 Fig), indicating that FLAG-tagged MoSET1 was functional.

**Fig 5:**
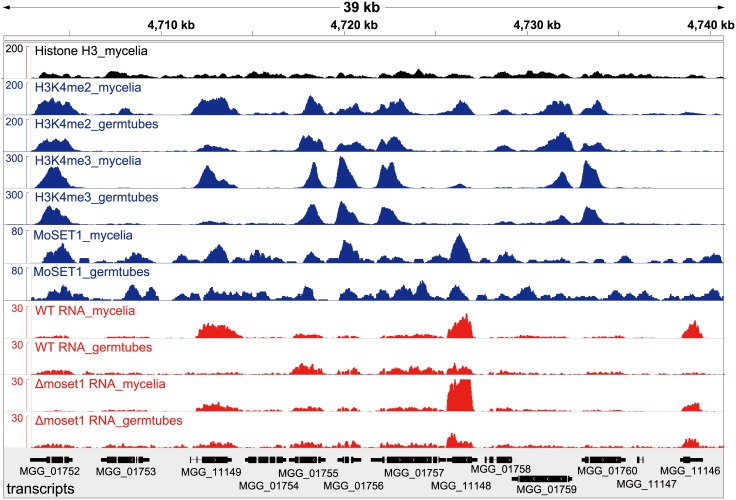
Representative view of ChIP-seq and RNA-seq results in *Magnaporthe oryzae* Chromosome 2. Blue and red peaks in the wiggle plot represents the normalized ChIP-seq and RNA-seq read coverage, respectively. Black peaks are for the control ChIP-seq library with antibodies against a C-terminal peptide of histone H3.

ChIP- and RNA-seq data were visualized by showing a representative chromosomal region (Fig 5). DNA immunoprecipitated with H3K4me2 and H3K4me3 antibodies predominantly localized to coding regions in the *M*. *oryzae* genome. As a control to show overall H3 levels, ChIP-seq data with antibodies against a C-terminal peptide of histone H3 was also presented (Fig 5). H3K4me3 accumulated relatively more in the 5′ gene regions as reported in other organisms (Figs 5 and S13) as reported in other organisms [39–41]. The number of genes showing differences in normalized mean coverage of H3K4me2 and H3K4me3 enrichment between vegetative mycelia and germination tubes is presented in Table 4. Genes with altered ChIP enrichment (*p* < 0.01), and over-represented Gene Ontology (GO) categories in the gene sets, are listed in S3 and S4 Tables. Changes in the H3K4me3 ChIP coverage were more frequently detected than those in the H3K4me2 coverage, suggesting that H3K4me3 could be a dynamic mark for gene regulation during infection-related morphogenesis in *M*. *oryzae*.

**Table 4:** Number of genes differentially enriched for H3K4me2/H3K4me3 in ChIP-seq analysis and differentially expressed in RNA-seq analysis between mycelia and germination tubes.

Strain	antibody	Myc > GT	Myc < GT
**ChIP-seq**			
WT	H3K4me2	293	133
WT	H3K4me3	223	398
Δmoset1-TF*	FLAG(MoSET1)	480	260
**RNA-seq**			
WT	-	2,141 (883)**	1,936 (1,201)**
Δmoset1	-	1,926	1,817

WT, wild-type strain Br48; Myc, mycelia; GT, germination tubes

The edgeR package was used to identify genes differentially expressed or enriched with the corrected p-value cutoffs of 0.01 (ChIP-seq analysis) or 0.001 (RNA-seq analysis).

*, Data from twoΔmoset1 complemented strains (Δmoset1-TF2 and -TF3) with FLAG-tagged MoSET1 were used in the analysis

**, number in parentheses represent the number of MoSET1-dependent genes (see text)

MoSET1 ChIP-seq reads were also largely mapped to gene regions. In contrast to H3K4 methylation that showed specific enrichment patterns among genes, MoSET1 appeared to be distributed rather ubiquitously to almost every gene, albeit at varying levels. In Table 4, the number of genes showing differences in MoSET1 enrichment between vegetative mycelia and germination tubes is presented. In some cases such as MGG_11148 and MGG_11149, relative enrichment of H3K4 methylation in mycelia than in germination tubes was associated with levels of MoSET1 enrichment at the loci (Fig 5). Consistently, in 91 of 133 (68.4%) and 268 of 399 (67.2%) genes showing significant enrichment of H3K4me2 and H3K4me3, respectively (Table 4), normalized mean coverage of MoSET1 was concomitantly increased in germination tubes. However, it is not likely that different levels of H3K4 methylation among genes were simply attributed to levels of MoSET1 localization to their loci. For instance, while similar levels of MoSET1 coverage were observed at the MGG_01752 and MGG_01753 loci in germination tubes, much higher H3K4me2/me3 coverage was detected at the MGG_01752 locus than at the MGG_01753 locus (Fig 5). These results implied that MoSET1 could principally distribute throughout the genome but might not be always enzymatically active.

**Fig 6:**
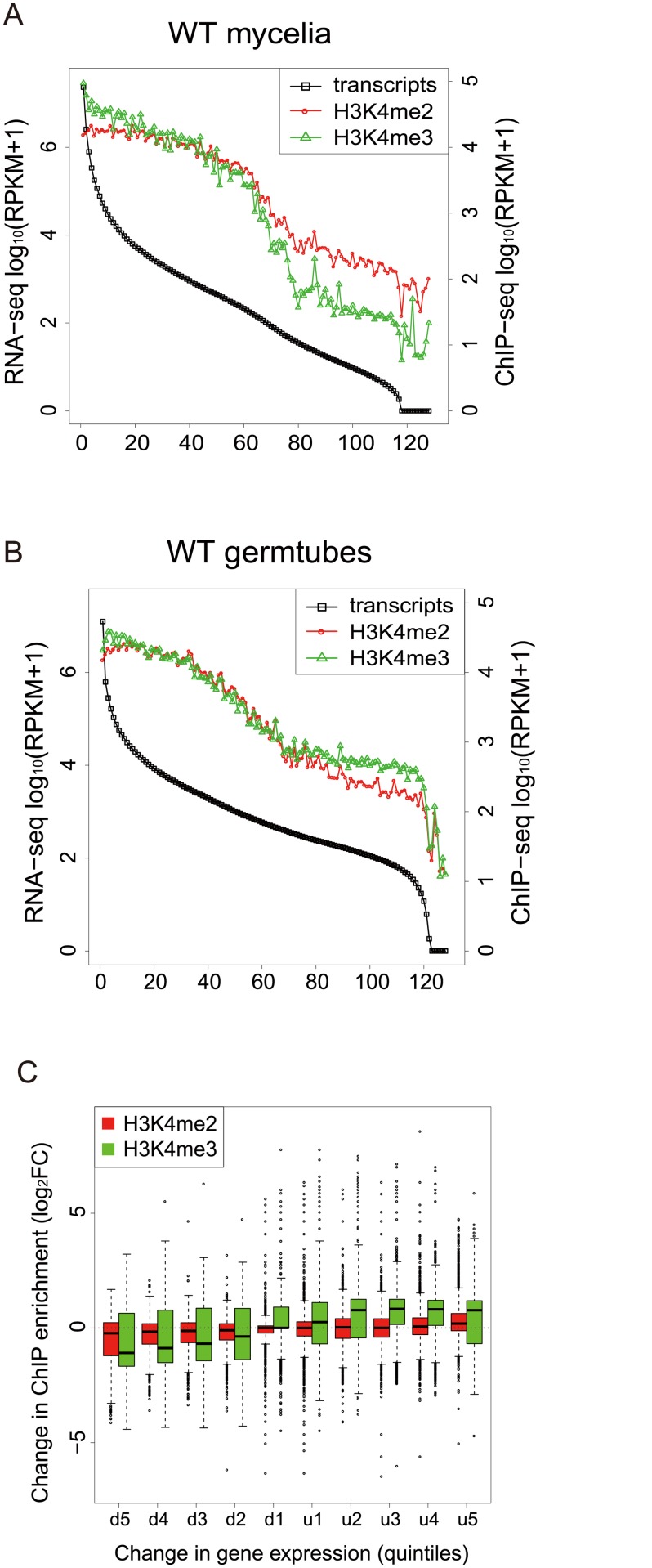
H3K4-me2 and -me3 abundance was generally associated with active transcription. (A)-(B) Genes were separated into groups of 100 genes according to their expression levels from high to low (left to right on the x axis) in mycelia (A) and germtubes (B). The average expression level of each gene group is given by an open black box. Open red circles and green triangles indicate the levels of H3K4 me2 and H3K4me3 in the gene groups, respectively. (C) Genes that were up- or down-regulated during infection-related morphogenesis were categorized into 10 groups (5 groups each in up-regulated [u1–u5] or down-regulated genes [d1–d5]) according to their change in transcript levels. The change in the H3K4 methylation (H3K4me2 or H3K4me3) in each group is represented as a box plot. In box plots, the central black line indicates the median value, and the top and bottom edges of the box are the 75th and 25th percentiles. The whiskers extend to the most extreme data points which are no more than 1.5 times the interquartile range from the box.

To gain a global view of the relationship between H3K4 methylation and gene expression, the *M*. *oryzae* genes were categorized into groups of 100 genes based on their expression levels, and the levels of H3K4 methylation in the gene groups were analyzed. Levels of H3K4me2 and H3K4me3 decreased as RNA levels decreased in mycelia and germination tubes (Fig 6A and 6B), indicating that H3K4 methylation was associated with active gene expression in *M*. *oryzae* in a similar way as reported in other organisms [39–41].

Next, we examined whether changes in the H3K4me2/me3 patterns were associated with gene activation or silencing during infection-related morphogenesis in *M*. *oryzae* at a global scale. RNA-seq analysis revealed that a total of 4,077 genes showed significant increases (1,936 genes) or decreases (2,141 genes) (*p* < 0.001) in expression levels in germination tubes (Table 4). These were sorted into five up-regulated and five down-regulated gene groups, and changes in H3K4me2/me3 ChIP enrichment in the groups were plotted (Fig 6C). The median H3K4me2 levels slightly decreased as the magnitude of transcript reductions increased. The H3K4me3 profile showed a more dynamic correlation with the transcript levels compared with the H3K4me2 levels, and the medians of the H3K4me3 levels were higher in the up-regulated gene groups and lower in the down-regulated gene groups.

It is noteworthy that the transcriptional activity of a gene was not always associated with local enrichment of H3K4 methylation as reported previously [18]. Higher H3K4me2/me3 coverage in mycelia than in germination tubes was accompanied with higher gene expression in some cases such as MGG_11149 in Fig 5. The up-regulation of the MGG_11149 gene in mycelia was significantly diminished in the*Δmoset1* mutant, supporting the idea that H3K4me2/me3 contributes to gene activation. However, transcriptional activation of the neighbor genes (MGG_01755, MGG_01756, and MGG_01757) in germination tubes did not concomitantly occur with apparent H3K4me2/me3 enrichment at their loci (Fig 5). In addition, with the MGG_11148 gene, H3K4me2/me3 enrichment in the wild-type strain appeared to be accompanied with gene activation but a similar change in gene expression also occurred in the*Δmoset1* mutant (Fig 5). Such apparent discrepancies were observed in many other cases. For example, H3K4me3 were significantly enriched in 398 genes and depleted in 223 genes in germination tubes compared to in mycelium (Table 4). Increase and decrease in RNA-seq read coverage were not accompanied with the H3K4me3 enrichment and depletion in 70 (17.6%) and 59 (26.5%) genes, respectively. Thus, H3K4 methylation tends to be overall associated with transcriptionally active genes, but the mechanism of gene regulation by H3K4 methylation is fairly complex, and possibly affected by other histone modifications.

### MoSET1 plays a role in gene activation and repression

The roles of MoSET1 in gene regulation were investigated by RNA-seq analysis of the *Δmoset1* mutant during infection-related morphogenesis. A total of 2,572 genes were differentially expressed in *Δmoset1* mycelia compared with the wild-type strain (*p* < 0.01), with 1,491 genes up-regulated and 1,081 down-regulated. Similarly, in germination tubes, 1,388 genes were up-regulated and 1,044 genes down-regulated in the *Δmoset1* mutant. These results indicated that a significant amount of *M*. *oryzae* genes were affected by the *Moset1* mutation. Interestingly, the number of genes up-regulated in the *Δmoset1* mutant was comparable to, or even more than, the number of down-regulated genes, suggesting that MoSET1 directly or indirectly plays a role in gene repression, as well as in gene activation.

**Fig 7:**
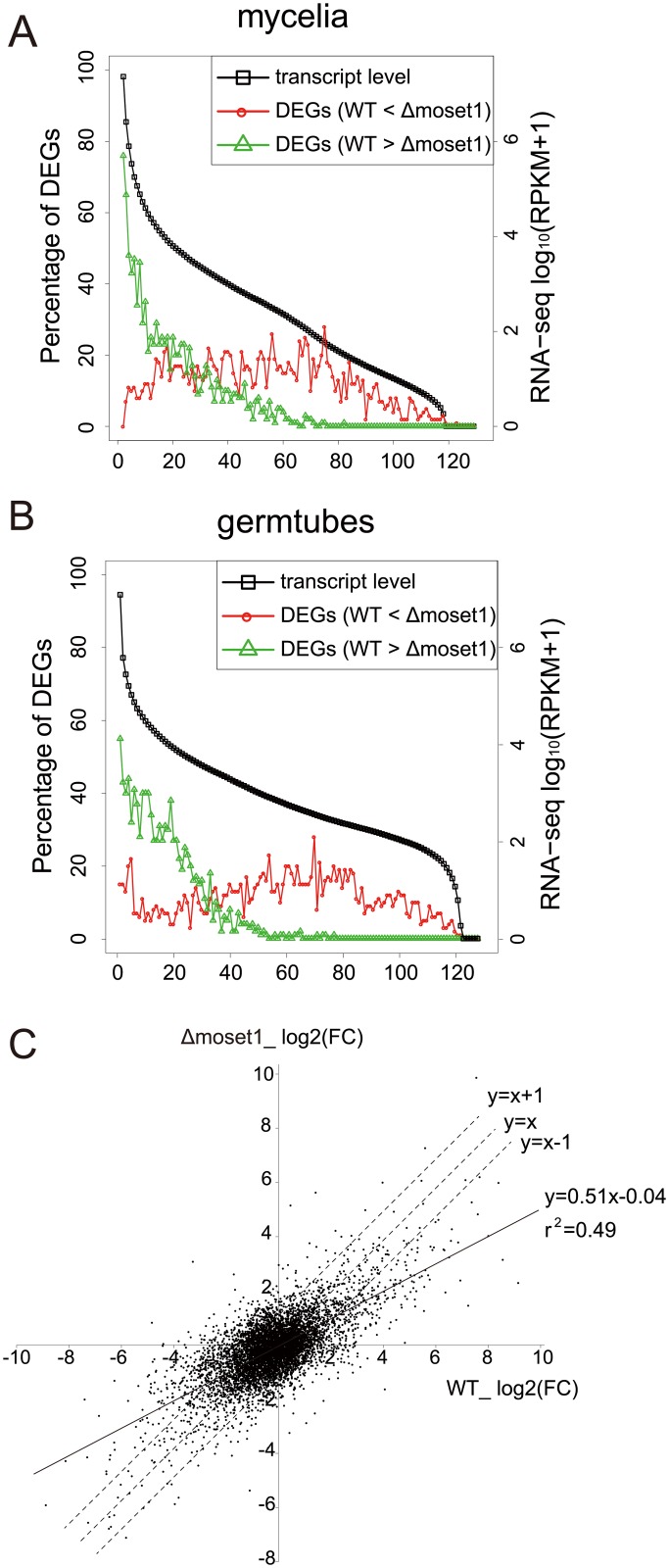
MoSET1 contributes to bilateral and global regulation of differentially expressed genes (DEGs). (A-B) Genes were separated into groups of 100 genes according to their expression levels from high to low (left to right on the x axis) in wild-type mycelia (A) and germtubes (B). Red open circles and green open triangles indicate the percentages of the down- and up-regulated DEGs between the wild-type (WT) and *Δmoset1* strains in the gene groups, respectively. (C) A log2 scale scatter plot comparison of fold changes (FC) in gene expression (germtubes/mycelia) between the WT and *Δmoset1* strains. A subset of 6,254 genes transcribed above 15.0 RPKM either in mycelia or germtubes of WT were plotted in the graph.

To analyze the characteristics of differently expressed genes between the wild-type and *Δmoset1* strains, we examined the frequency distribution of genes belonging to these gene groups in mycelia (Fig 7A) and germination tubes (Fig 7B), based on the expression levels in the wild-type strain. In both mycelia and germination tubes, genes down-regulated in the *Δmoset1* strain were highly biased to the high expression gene groups in the wild-type strain, while those up-regulated in the *Δmoset1* strain were more frequently distributed in medium and low expression level gene groups (Fig 7A and 7B).

We next addressed how the *moset1*mutation affected gene regulation by comparing the fold change (FC) in gene expression during infection-related morphogenesis between the wild-type and*Δmoset1* strains. A log_2_-scale scatter plot showed a positive linear correlation between the FC values in the wild-type and*Δmoset1* strains (*p* = 6.8E-06) (Fig 7C). However, regression analysis gave the equation, y = 0.51x-0.04 with the correlation coefficient, r^2^ = 0.49, indicating that the correlation was only moderate. The slope lower than one indicated that gene expression changes were generally more marked in the wild-type strain (x-axis) than in the*Δmoset1* mutant (y-axis) for both up- and down-regulated genes (Fig 7C). This supported the conclusion that MoSET1 contributed to bilateral and global gene regulation during infection-related morphogenesis in *M*. *oryzae*.

### Characterization of differentially expressed genes (DEGs) during infection-related morphogenesis in a *Moset1*-depedent or -independent manner

To assess the effect of MoSET1 on gene induction and repression during infection-related morphogenesis, we focused on a subset of 4,077 genes that showed a significant change in expression levels between wild-type mycelia and germination tubes in the RNA-seq analysis (Table 4). There were less up-regulated genes in the subset (1,936 genes) than down-regulated genes (2,141 genes). To understand the dependency of their gene expression on *Moset1*, we defined the criterion of “*Moset1*-dependent genes” based on a comparison of FC values (germination tubes/mycelia) between wild-type and *Δmoset1* strains. When the rate of FC increase or decrease of a gene in the *Δmoset1* strain was less than 50% of the wild-type strain, the gene was categorized as a *Moset1*-dependent gene. Genes not meeting the criterion were classified as “*Moset1*-independent genes”. Based on these criteria, 1,201 and 735 genes were categorized as *Moset1*-dependent and -independent up-regulated genes, respectively; and 883 and 1,258 genes were grouped as *Moset1*-dependent and -independent down-regulated genes, respectively. Therefore, approximately half of the transcriptional changes during infection-related morphogenesis were directly or indirectly dependent on *Moset1* in *M*. *oryzae*. Dependency on *Moset1* was more evident with the up-regulated genes. Lists of the *Moset1*-dependent and -independent genes and over-represented GO categories in the gene sets were given in S5 and S6 Tables, respectively.

**Fig 8:**
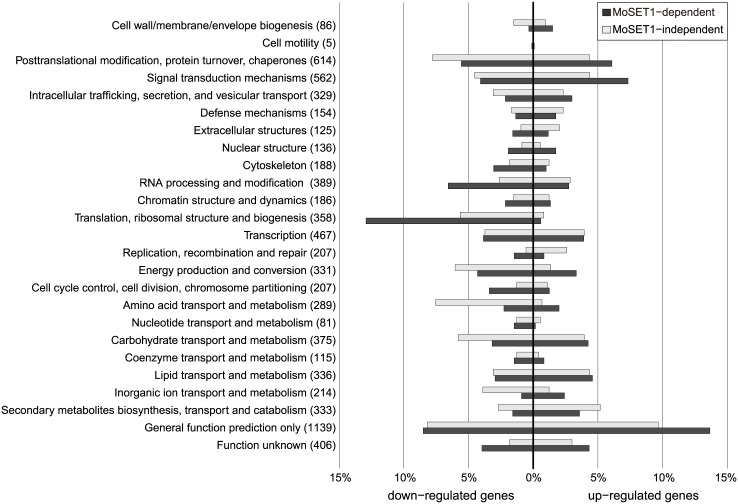
KOG functional classification of differentially expressed genes (DEGs) between vegetative mycelia and germtubes with regard to dependency on *Moset1*. A total of 4,077 DEGs identified in the RNA-seq analysis were assigned to KOG functional categories. The assigned DEGs were further subdivided into four groups based on the direction of regulation (up or down) and the dependency on *Moset1* (see text). The percent distribution of DEGs in the subgroup by KOG category is presented. The number of *M*. *oryzae* genes belonging to each KOG functional category is shown in parentheses.

*Moset1*-dependent and -independent genes were further classified using euKaryotic Orthologous Group (KOG) functional categories (Fig 8). The KOG category “signal transduction mechanisms” was highly over-represented in the *MoSET1*-dependent up-regulated gene set. Consistently, several GO categories related to “signal transduction mechanisms” were significantly over-represented in the gene set (S6 Table). In this category, forty one kinases and thirteen GTPase regulators were detected, indicating that a large number of key signal mediators were transcriptionally regulated by MoSET1, either directly or indirectly. Interestingly, twenty-four active transmembrane transporters, including MgAPT2 (MGG_02767), MgAPT3 (MGG_04066), and MgAPT4 (MGG_04852) [42] were regulated by *MoSET1*.

The KOG categories over-represented in the *Moset1*-dependent down-regulated gene set were different from those in the upregulated gene sets, and included “translation, ribosomal structure and biogenesis” and “RNA processing and modification”. Interestingly, sixty-four structural constituents of the ribosome were found in this criterion (S6 Table), indicating that MoSET1 was associated with down-regulation of a significant portion of ribosome-related genes. In addition, various nucleic acid binding proteins, especially those that bind RNA, were down-regulated in a *Moset1*-dependent manner. These included proteins homologous to nuclear ribonucleoprotein, RNA helicase, tRNA synthetase, rRNA biogenesis protein, poly(A) polymerase, and poly(A)-binding protein.

**Fig 9:**
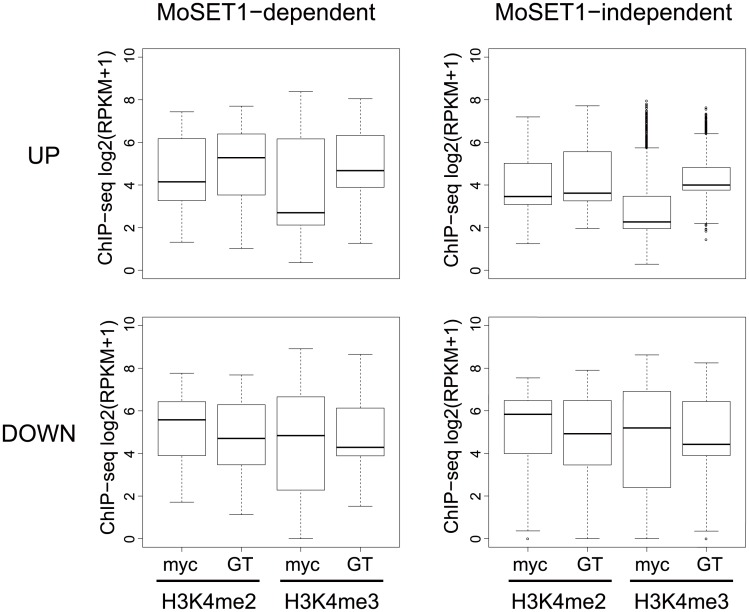
Comparisons of H3K4 methylation dynamics during germination tube formation between MoSET1-dependent and -independent gene sets. “MoSET1-dependent genes” were defined based on a comparison of FC values (germination tubes [GT]/mycelia [myc]) between wild-type and Δmoset1 strains (see Results). When the rate of FC increase or decrease of a gene in the Δmoset1 strain was less than 50% of the wild-type strain, the gene was categorized as a MoSET1-dependent gene. Genes not meeting the criterion were classified as “MoSET1-independent genes”. MoSET1-dependent and -independent genes were further divided into “up-regulated (UP)” and “down-regulated (DOWN) gene groups. The levels of H3K4 methylation (H3K4me2 or H3K4me3) in each group is represented as a box plot. In the box plots, the central black line indicates the median value, and the top and bottom edges of the box are the 75th and 25th percentiles. The whiskers extend to the most extreme data points which are no more than 1.5 times the interquartile range from the box.

Finally, we addressed whether *Moset1*-dependent gene regulation is directly related to H3K4 methylation. Levels of H3K4me2 and H3K4me3 enrichment in mycelia and germination tubes were plotted separately by the four gene criteria indicated in Fig 9. In the up-regulated gene groups, levels of H3K4 methylation were generally higher in the *Moset1*-dependent genes than in the *Moset1*-independent genes. In addition, the *Moset1*-dependent genes showed stronger enrichment of H3K4me2 in germination tubes, where they were up-regulated, than did the *Moset1*-independent genes (Fig 9). Thus, in the up-regulated gene groups, changes in H3K4 methylation were more dynamic in the *Moset1*-dependent genes than in the *Moset1*-independent genes, suggesting direct contribution of H3K4 methylation to gene activation.

In contrast, in the down-regulated gene groups, while both H3K4me2 and H3K4me3 levels decreased in germination tubes, where the genes in these criteria were down-regulated, only slight difference was observed in H3K4me2 and H3K4me3 enrichment patterns between the *Moset1*-dependent and -independent genes. This may suggest that the *Moset1*-dependency in the down-regulated genes was not directly resulted from H3K4 methylation.

## Discussion

### MoSET1 plays the most crucial role in infection-related morphogenesis among the eight *M*. *oryzae* KMT genes examined

The gene knockout studies of the eight KMT genes in *M*. *oryzae* revealed that MoKMT1, MoSET1, MoKMT3, MoKMT6, and MoKMT2H played significant roles in infection-related morphogenesis and/or pathogenicity to varying degrees, while MoKMT4, MoKMT5, and MoSET6 did not. *Δmokmt1* mutants did not display detectable defects in infection-related morphogenesis, but showed a slight reduction in vegetative growth and of pathogenicity on host plants. MoKMT1 belongs to the KMT1 family responsible for methylation at H3K9 and is paralogous to *N*. *crassa* DIM-5. In *N*. *crassa*, H3K9me3 catalyzed by DIM-5 is recognized by HP1 that forms a complex with DIM-2 DNA methyltransferase [9]. HP1 is a structural protein essential for heterochromatin formation, and leads to gene repression [43]. Since H3K9me3 is a conserved epigenetic mark for gene repression, MoKMT1 is likely involved in gene repression in *M*. *oryzae*. Interestingly, *Δmokmt6* mutants showed a reduction in pathogenicity at levels similar toΔmokmt1 (Tables 2 and 3). The KMT6 family enzymes catalyze H3K27 methylation, a mark for gene repression. In *F*. *graminearum*, a KMT6 deletion mutant exhibited developmental defects and reduced pathogenicity as did*Δmokmt6* mutants [18]. Similarly, in the plant pathogenic fungus, *Leptosphaeria maculans*, silencing of LmDIM5 belonging to the KMT1 family resulted in a reduction in pathogenicity [20]. Thus, gene repression itself or proper switching from gene repression to expression may be required for the full pathogenicity of the fungi, or these KMT genes may have functions other than gene repression.

*Δmokmt3* and*Δmokmt2h* mutants showed a significant reduction in vegetative growth, germination, appressorium formation, conidiation, and pathogenicity to host plants. Thus, these mutants had defects in every phenotypic assay performed in this study. The reduction in pathogenicity was more severe in*Δmokmt2h* mutants than in*Δmokmt3* mutants. MoKMT3 is paralogous to *N*. *crassa* SET-2, which belongs to the KMT3 family responsible for methylation at H3K36. H3K36me3 levels peak within the body of active genes, and may be associated with transcription elongation through contributing to the maintenance of chromatin architecture [44, 45]. In *N*. *crassa*, SET-2 loss-of-function mutants show various defects, including slow vegetative growth, low conidiation, and female sterility [16]. This is consistent with our results. Thus, MoKMT3 possibly affected the correct expression of a number of genes involved in phenotypic defects.

While possible paralogs of MoKMT2H are widely conserved in ascomycete fungi, their biological roles have not been well-characterized. Based on phylogenetical analysis, the most closely related KMT to MoKMT2H in mammals is ASH1L, which is implicated in H3K4 and H3K36 methylation, and in transcriptional activation of certain genes, including Hox genes [46, 47]. Therefore, if MoKMT2H is a functional homolog of ASH1L, it follows that MoKMT2H may also contribute to gene activation in *M*. *oryzae*.

MoSET1 was among the most crucial KMTs for infection-related morphogenesis and symptom development on host plants in *M*. *oryzae*. *Δmoset1* mutants showed severe defects in vegetative growth, appressorium formation, conidiation, and pathogenicity to host plants, but not in the rate of germination. MoSET1 catalyzes methylation at H3K4, an evolutionary conserved epigenetic mark for gene activation. Our data suggested that this epigenetic mark could be the most important histone methylation for infection-related gene expression in *M*. *oryzae*. RNA-seq analysis of the *Δmoset1* and wild-type strains suggested that approximately half of the genes induced or repressed during infection-related morphogenesis were dependent on *MoSET1*.

*Moset1*-dependent genes appeared to be involved in various infection-related processes. One such process is appressorium formation. cAMP signaling is crucial for appressorium formation in *M*. *oryzae* [36, 37]. Several genes involved in the cAMP signaling pathway leading to appressorium formation, including MacI (MGG_09898), MacI-interacting protein (MGG_05531), Mck1 (MGG_00883), and Sum1 (MGG_07335) were categorized as *Moset1*-dependent up-regulated genes [48]. The deficiency in transcriptional up-regulation of these genes in the germination tubes may be the cause of severe reduction in appressorium formation in the *Δmoset1* mutants. This assumption is consistent with appressorium formation in the mutants being restored by exogenous cAMP.

Pathogenicity of *Δmoset1* mutants to host plants was not recovered by cAMP addition, indicating that *Δmoset1* mutants had defects in pathogenicity other than appressorium formation. A large number of transporters were up-regulated in a *Moset1-*dependent manner in germination tubes (Fig 8). One such gene, MgApt2 is a P-type ATPase involved in exocytotic processes during plant infection [42]. Exocytotic mechanisms are involved in the delivery of proteins into plant cells to suppress plant defenses.

Signal transducers were highly enriched in the *Moset1*-dependent gene set. MgATG1 (MGG_06399), a serine/threonine-protein kinase found in this group, is involved in autophagy and the generation of normal turgor pressure in the appressorium, and is thus essential for successful infection [49]. However, many important protein kinases for fungal pathogenesis, including Pmk1 (MGG_09565), CpkA (MGG_06368), Msp1 (MGG_05344), and MST7 (MGG_00800) were not *Moset1*-dependent genes. Thus, not all signal transducers responding environmental stimuli required MoSET1 for their activation.

Cell-wall degradation enzymes (CWDEs) are possible *Moset1*-dependent contributors to the full pathogenicity of the fungus. We previously reported that CWDEs, such as GH7 and GH8 cellulases and GH10 and GH11 xylanases, were greatly activated during infection [50, 51]. Their gene activation was often induced by presence of relevant cellulose or xylan substrates. Substrate-dependent gene activation of the cellulases was severely compromised in the *Δmoset1* [31]. Therefore, lack of CWDE activation during infection may be the cause of the severe reduction observed in the pathogenicity of the *Δmoset1* mutants on the host plants. Gene activation of CWDEs was, however, barely detectable in the RNA-seq analysis; this is a consequence of RNA being extracted from germination tubes on slide glasses, thus having no available enzyme substrates.

It should be note that, since*Δmoset1* mutants were also impaired in vegetative growth, the role of MoSET1 in gene regulation was not infection specific. Thus, the down-regulation of such general genes could also contribute to the loss of pathogenicity in*Δmoset1* mutants.

### *Moset1* is involved in positive and negative gene regulation during infection-related morphogenesis in *M*. *oryzae*

In eukaryotes, H3K4 methylation is an epigenetic mark for gene activation. ChIP-seq or ChIP-chip analysis together with transcriptome analysis in human, *Arabidopsis*, and *S*. *cerevisiae* revealed a global positive correlation between H3K4me2/me3 and active transcription [39–41]. In *M*. *oryzae*, substrate-induced gene expression of GH6 and GH7 cellulases was associated with enrichment of H3K4me2 [31]. Interestingly, however, expression levels of GH6 and GH7 cellulases under non-inducing conditions increased in the *Δmoset1* mutant, suggesting a possible role of H3K4 methylation in gene repression [31]. This is consistent with the observations in *Aspergillus nidulans* and *A*. *fumigatus*, where a deletion mutant of the *CclA* gene, which encodes a component of the COMPASS complex catalyzing H3K4 methylation, results in a reduction in H3K4me2 and H3K4me3, and also causes increased gene expression of cryptic SM gene clusters [21, 22]. Thus, *CclA*-mediated H3K4 methylation appears to contribute to gene silencing of SM clusters. It is noteworthy that SET1 in *S*. *cerevisiae* was originally identified as a gene required for transcriptional silencing of silent mating-type loci in the subtelomeric region [10]. Subsequently, SET1 was demonstrated to play a role in silencing rDNA and the retrotransposon Ty1 in *S*. *cerevisiae* [23, 24]. Consistently, contribution of MoSET1 to the repression of ribosome-related genes was shown in this study (Fig 8). Recently, SET-1 was reported to have a role in DNA methylation of the frq promoter in *N*. *crassa* [52]. Thus, SET1 orthologs in fungi are involved in gene silencing in addition to gene activation.

Our RNA-seq analysis revealed that significant numbers of *M*. *oryzae* genes were up- or down-regulated in the *Δmoset1* mutant in comparison with the wild-type strain, supporting that H3K4 methylation is directly or indirectly involved in both gene activation and repression. *Moset1*-dependent gene up-regulation was largely detected in highly-expressed genes in the wild-type strain, whereas *Moset1*-dependent gene repression was more frequently observed in genes with middle or low expression levels (Fig 7). It is to be noted that the roles of *Moset1*-dependent gene repression in the pathogenicity of the fungus were so far unclear while *Moset1*-dependent gene activation, most likely, indeed contributed to infection-related morphogenesis in *M*. *oryzae* as discussed above.

Apparent differences in the roles of KMT2 proteins among organisms can be attributed to the experimental approaches. In fact, this study demonstrates that results obtained by ChIP-related techniques in fungi are not much different from those obtained in higher eukaryotes. The role of SET1 orthologs in gene repression has mainly been revealed by gene knock-out approaches that are often used in fungi but seldom in higher eukaryotes. In higher eukaryotes, several SET1 homologs redundantly serve as catalytic enzymes for methylation at H3K4. At least six Set1 homologs (Set1A, Set1B, MLL1, MLL2, MLL3, and MLL4) have been identified in mammalian cells, making gene knock-out strategies ineffective. Thus, it might be possible that the complete loss of SET1 homologs in the genome uncovered their additional functions that were hard to find by other approaches. However, it also should be noted that, in gene knockout approaches, it is difficult to distinguish direct and indirect effects of the loss of the target gene. Since SET1 homologs positively regulate global gene expression, their knockout mutants might fail to activate genes required for proper gene repression. Thus, de-repression of genes in the*Δmoset1* mutant may not arise directly from the loss of the gene but may come from secondary causes, for example, insufficient expression of repressor genes in the mutant. Our results showed that changes in H3K4 methylation during germination tube formation were more dynamic in the *Moset1*-dependent gene set than in the *Moset1*-independent gene set among the up-regulated genes but not among the down-regulated genes (Fig 9), suggesting that the *Moset1*-dependency in the down-regulated genes could not be directly related to changes in H3K4 methylation. The data favors the hypothesis that the *Moset1*-dependent gene repression arose from indirect effects of the loss of MoSET1 even though other hypotheses are not completely eliminated. For example, recently, it has been reported that H3K4 monomethylation functioned as a mark for gene repression in several types of mammalian cells [25]. Thus, it might be possible that the lack or severe depletion of H3K4 monomethylation in the set1 or cclA deletion mutants resulted in activation of genes that were repressed under the wild-type background.

## Materials and Methods

### Strains and growth conditions

The wheat-infection *M*. *oryzae* isolate, Br48 [53] and its transformants constructed in this study (S1 Table) were kept on barley seeds media at 4°C for long-term storage [54]. For working culture, a barley grain from the stock culture was placed on a PDA (potato dextrose agar) slant media and cultured at 25°C. Fungal plugs were transferred to flasks containing complete medium (5% sucrose, 3% casamino acids, and 3% yeast extract) and incubated in a shaker at 120 rpm at 25°C for 4 days. To prepare conidial suspension, fungal strains were cultured on oatmeal agar plates (40g of oatmeal, 17g of agar in 1000 ml water) in the darkness at 25°C for 5 days. Then, aerial mycelia were removed by rubbing surface of mycelia with a sterile microtube, and further incubated under BLB light for 3 days at 25°C to induce conidiation.

### Construction of *M*. *oryzae* gene knock-out mutants

In this study, a split-marker gene disruption strategy [32] was used to obtain a gene knock-out mutant in *M*. *oryzae* (see S1–S7 Figs). First, PCR products of the upstream and downstream of a targeted gene were cloned separately into the multiple cloning site of pSP72-hph that carries the Hygromycin resistance gene cassette [55]. Primers used in this study are given in S7 Table. PCR fragments amplified from the resulting 5’ and 3’ constructs were mixed and introduced into fungal spheroplasts by a polyethylene glycol (PEG)-mediated method as previously described [56]. For initial screening, colonies PCR were performed with appropriate sets of primers for each gene. The candidate strains were further examined by Southern blot analysis. Fungal genomic DNA was extracted using Plant Genomic DNA Extraction Miniprep System (Viogene) following the manufacturer’s instruction. Southern blot analysis was performed using the DIG DNA Labeling and Detection Kit (Roche Applied science). Ten to twenty micrograms of genomic DNA were digested by appropriate restriction enzymes. The digests were separated by agarose gel electrophoresis and transferred to Hybond N^+^ (Amersham biosciences). The hybridization procedures were carried out according to the manufacturer’s instructions. The positions of DIG-labeled probes used in Southern blot analysis are given in S1–S7 Figs.

Genetic complementation of KMT deletion mutants was performed by introducing the corresponding native genomic fragment to them. Genomic DNA fragments containing KMT genes with their 5’flanking and 3’flanking regions were amplified with pairs of specific primers (S7 Table) using KOD FX Neo (Toyobo), and cloned into pBluescript SK(+). Each of the resulting plasmids was introduced into the corresponding KMT mutant with pII99 carrying the geneticin-resistance gene.

### Phenotypic characterization of *M*. *oryzae* knock-out mutants

Growth rates (colony diameter) of *M*. *oryzae* mutants on PDA media were measured up to 14 days with three replications. For conidiation assay, conidia were harvested 3 days after BLB induction by suspending them with 20ml sterile distilled water per plate. Spore concentration was estimated by microscopic observation of at least 20 visual fields using a hemocytometer. For conidial germination and appressorium formation assays, conidial suspension (10^5^ spores per ml) dropped on slide glasses was incubated in a humidity box at 25°C. The rates of conidia germination and appressorium formation were counted by microscopic observation of at least 200 spores after 5 and 24 h incubation at 25°C, respectively.

Infection assay was performed as described previously [57]. Wheat and barley seedlings were grown in vermiculite supplied with liquid fertilizer in plastic pots (5.5cm×15cm×10cm) at 22°C in a controlled-environment incubator with a 12h photoperiod for 8 days. The plant cultivars used were wheat cultivars “Norin 4”, “Chinese spring” and Thatcher, and barley cultivars “Russian No.74” and “Nigrate”. Conidia suspension (1–2☓10^5^ spores/ml) containing 0.01% Tween 20 was sprayed to the primary leaves of 8-day-old wheat and barley seedlings. The inoculated seedlings were maintained under high humidity and dark conditions for 24 hours then moved to an incubator at 22°C with a 12h photoperiod for 5 days. Symptoms appeared were assessed based on the size and color of lesions to determine infection type. The size of a lesion was rated from 0 to 5: 0, no visible evidence if infection; 1, pinpoint spots; 2, small lesion (<1.5 mm); 3, lesion with an intermediate size (<3 mm); 4, large and typical lesion; 5, complete blighting of leaf blades. Green (G) and brown (B) lesions were regarded as susceptible and resistant responses, respectively [58].

For microscope observation of host cell response to *M*. *oryzae*, inoculated leaves were picked up at 48 h post inoculation (hpi) and deeply boiled in alcoholic lactophenol (lactic acid/phenol/glycerol/distilled water/ethanol = 1:1:1:1, v/v/v/v) for 2 min as descried previously [57]. Samples were observed using an epifluorescence microscope under bright and fluorescent fields. Host response was classified into four types: no reaction, papilla formation, hypersensitive reaction (HR), and hyphal growth.

### Western blot analysis

Fungal mycelia powder was suspended in 1×TBS buffer (50 mM Tris-HCl [pH 7.5], 150 mM NaCl) containing 1% Nonidet P-40. The homogenates were centrifuged (12,000 rpm, 2 minutes), and supernatants were collected. Proteins in the supernatants were then heat-treated at 80°C for 10 min to precipitate contaminating proteins. The supernatant was recovered by centrifugation, and subjected to 15% SDS_polyacrylamide gel electrophoresis. After blotted to PVDF membrane, proteins were probed with the following primary antibodies; anti-H3K4me1 (Active motif #39298), anti-H3K4me2 (Active motif #39141), anti-H3K4me3 (Active motif #39159), anti-H3K9me3 (Active motif #39161), anti-H3K27me3 (Active motif #39535), anti-H4K20me3 (Active motif #39181) and a C-terminal peptide of histone H3 (Active motif #39163). Washing was performed three times with TBS-T buffer containing a higher concentration of NaCl (50 mM Tris-HCl [pH 7.5], 190 mM NaCl, 0.05% Tween 20). Proteins reacting with the primary antibodies were visualized by appropriate peroxidase (HRP)-conjugated secondary antibodies and ECL plus western blotting detection regents.

### Chromatin immuno-precipitation (ChIP) assays

An N-terminal 2xFLAG-tagged MoSET1 construct was made by PCR amplification with the primers, FLAG-MoSET1-F and MoSET1-TGA-R (S7 Table), and then fused to the native MoSET1 promoter and terminator sequences by In-Fusion HD cloning kit (Clontech Laboratories) at *Eco*RV site in pBluescript SK(+) with the primers, IF-PMoSET-F, IF-PMoSET-R, IF-TMoSET-F, and IF-TMoSET-R (S7 Table). The resulting construct was introduced into the *Δmoset1* mutant and used in chromatin immuno-precipitation (ChIP) analysis.

ChIP experiments were performed with germinating conidia and vegetative mycelia using the ChIP-IT Express kit (Active motif #53008) according to manufacturer's instructions using sonication as a method for chromatin shearing. In addition to the antibodies used in western blot analysis, Anti-DDDDK-tag mAb-magnetic beads (Medical & Biological Laboratories, Japan) was used in ChIP experiments. Briefly, samples (100mg) were treated with 1% formaldehyde by shaking gently (100rpm) for 30 minutes at room temperature. Chromatin was sheared on ice by sonication using a Bioruptor apparatus (Diagenode) for 3 cycles of 1 min on at high intensity (200 W) and 30 sec off, followed by 4 cycles of 1 min on at medium intensity (160 W) and 30 sec off. The size of the sheared chromatin was around 200 to 1,000 bp as determined by agarose gel electrophoresis. After immunoprecipitation with an appropriate antibody, DNA fragments were recovered by Proteinase K treatment. Indexed ChIP-seq libraries were prepared with the NEBNext ChIP-Seq Library Prep Master Mix Set for Illumina (New England Biolabs) according to the manufacturer’s instructions. Fragment size selection of ChIP-seq libraries was done using Agencourt AMPure XP beads (Beckman Coulter). The products were purified and enriched with PCR to create the final double stranded cDNA library. The MiSeq system (Illumina) was used to sequence the cDNA library.

### RNA extraction, cDNA library preparation and sequencing

RNA extraction and cDNA preparation were carried out as described previously with a few modifications [31]. Total RNA was isolated using Sepasol RNA I Super (Nakalai Tesque), and used for cDNA synthesis using ReverTra Ace qPCR RT master mix with genomic DNA remover KIT (Toyobo). Depletion of rRNA was performed using the Ribo-Zero rRNA removal kit for human/mouse/rat (Epicentre). Indexed RNA-seq libraries were prepared with the NEBNext Ultra™ RNA Library Prep Kit for Illumina kit (New England Biolabs) or NEXTflex™ Directional RNA-Seq Kit (BIOO Scientific Corp.) according to the manufacturer’s instructions. Fragment size selection of RNA-seq libraries was done using Agencourt AMPure XP beads. The products were purified and enriched with PCR to create the final double stranded cDNA library. The MiSeq system (Illumina) was used to sequence the cDNA library.

### NGS data analysis

The RNA-seq and ChIP-seq reads (75–120 bp) were mapped to the genome of the *Magnaporthe oryzae* strain 70–15 (release 8.0, http://www.broadinstitute.org/) using TopHat v2.0.10 [59] and bwa v0.6.2-r126 [60], respectively. RNA-Seq and ChIP-seq data were visualized in the Integrative Genomics Viewer genome browser [61]. The edgeR package [62] for R v3.0.1 [63] was used for TMM normalization [64], identification of differentially expressed genes (DEGs) from RNA-seq data, and detection of genes differentially enriched for histone modifications from ChIP-seq data with the corrected *p*-value [65] cutoffs of 0.01 (ChIP-seq analysis) or 0.001 (RNA-seq analysis). The GOstats package [66] was used to identify statistically significant enriched Gene Ontology (GO) categories.

## Supporting Information

S1 Fig Construction of *Mokmt1* deletion strains (Δ*mokmt1*). (**A**) Schematic representation of the *Mokmt1* (MGG_06852) locus in the wild type (Br48) and *Δmokmt1* strains. The map position of *Mokmt1* is from 2,991,740 to 2,993,241 on chromosome supercont8.1 of *Magnaporthe oryzae* 70–15. Deletion strains were constructed using the split-marker system with primers *Hmt18up-XhoI-F/HY* and *YG/Hmt18down-Bl*g*II-R*. A 214-bp probe (red bar) was amplified by polymerase chain reaction with *Hmt18down-KpnI-F/Hmt18-sreR* and used for Southern blot analysis. *Pst*I-digestion of genomic DNA from Br48 and Δmokmt1 is expected to generate 2360-bp and 917-bp DNA fragments, respectively. (**B**) Southern blot analysis using the probe and *Pst*I-digested genomic DNA. MoKMT1E indicates an ectopic mutant.
(PDF)

S2 Fig Construction of *Mokmt3* deletion strains (Δ*mokmt3*). (**A**) Schematic representation of the *Mokmt3* (MGG_01661) locus in the wild type (Br48) and *Δmokmt3* strains. The map position of *Mokmt3* is from 4,375,150 to 4,379,185 on chromosome supercont8.2 of *Magnaporthe oryzae* 70–15. Deletion strains were constructed using the split-marker system with primers *27up-F/HY* and *YG/Hmt27-1661down-BglII-R*. A 429-bp probe (red bar) was amplified by polymerase chain reaction with *Probe27-F/Probe27-R* and used for Southern blot analysis. *Cla*I-digestion of genomic DNA from Br48 and **Δ** *mokmt3* is expected to generate 3968-bp and 3056-bp DNA fragments, respectively. (**B**) Southern blot analysis using the probe and *Cla*I-digested genomic DNA. MoKMT3E indicates an ectopic mutant.
(PDF)

S3 Fig Construction of *Mokmt4* deletion strains (Δ*mokmt4*). (**A**) Schematic representation of the *Mokmt4* (MGG_05254) locus in the wild type (Br48) and *Δmokmt4* strains. The map position of *Mokmt4* is from 5,164,077 to 5,166,276 on chromosome supercont8.3 of *Magnaporthe oryzae* 70–15. Deletion strains were constructed using the split-marker system with primers Hmt22-05254-SphI-up-F/HY and YG/22down-R. A 491-bp probe (red bar) was amplified by polymerase chain reaction with H22probe-F/H22probe-R and used for Southern blot analysis. *Sac*I-digestion of genomic DNA from Br48 and *Δmokmt4* is expected to generate 4592-bp and 3835-bp DNA fragments, respectively. (**B**) Southern blot analysis using the probe and *Sac*I-digested genomic DNA. MoKMT4E indicates an ectopic mutant.
(PDF)

S4 Fig Construction of *Mokmt5* deletion strains (Δ*mokmt5*). (**A**) Schematic representation of the *Mokmt5* (MGG_07393) locus in the wild type (Br48) and *Δmokmt5* strains. The map position of *Mokmt5* is from 42,3851 to 427,601 on chromosome supercont8.3 of *Magnaporthe oryzae* 70–15. Deletion strains were constructed using the split-marker system with primers *Set9up-F/HY* and *YG/Set9down-R*. A 391-bp probe (red bar) was amplified by polymerase chain reaction with *Set93down-F/Set9scree-R* and used for Southern blot analysis. *Cla*I-digestion of genomic DNA from Br48 and Δmokmt5 strains is expected to generate 3380-bp and 614-bp DNA fragments, respectively. (**B**) Southern blot analysis using the probe and *Cla*I-digested genomic DNA. MoKMT5E indicates an ectopic mutant.
(PDF)

S5 Fig Construction of *Mokmt6* deletion strains (Δmokmt6). (**A**) Schematic representation of the *Mokmt6* (MGG_00152) locus in the wild type (Br48) and *Δmokmt6* strains. The map position of *Mokmt6* is from 3,929,861–3,934,291 on chromosome supercont8.5 of *Magnaporthe oryzae* 70–15. Deletion strains were constructed using the split-marker system with primers Mgg-00152 up-F/HY and *YG/Mgg-00152 down-R*. A 463-bp probe (blue bar) was amplified by polymerase chain reaction with *Mgg-00152probe-F/Mgg-00152probe-R* and used for Southern blot analysis. *Nde*I-digestion of genomic DNA from Br48 and *Δmokmt6* strains is expected to generate 1986-bp and 1667-bp DNA fragments, respectively. (**B**) Southern blot analysis using the probe and *Nde*I digested genomic DNA. MoKMT6E indicates an ectopic mutant.
(PDF)

S6 Fig Construction of *Mokmt2h* deletion strains (Δ*mokmt2h*). (**A**) Schematic representation of the *Mokmt2h* (MGG_02937) locus in the wild type (Br48) and *Δmokmt2h* strains. The map position of *Mokmt2h* is from 941,917 to 946,513 on chromosome supercont8.7 of *Magnaporthe oryzae* 70–15. Deletion strains were constructed using the split-marker system with primers 23up-HindIII-F/HY and YG/22down-BglII-R. A 508-bp probe (red bar) was amplified by polymerase chain reaction with Hmt23-srce-F/23up-SphI-R and used for Southern blot analysis. *Pvu*II-digestion of genomic DNA from Br48 and *Δmokmt2h* strains is expected to generate 5244-bp and 3970-bp DNA fragments, respectively. (**B**) Southern blot analysis using the probe and *Pvu*II-digested genomic DNA.
(PDF)

S7 Fig Construction of *Moset6* deletion strains (Δ*mokset6*). (**A**) Schematic representation of the *Moset6* (MGG_15522) locus in the wild type (Br48) and *Δmoset6* strains. The map position of *Moset6* is from 494,343 to 496,716 on chromosome supercont8.2 of *Magnaporthe oryzae* 70–15. Deletion strains were constructed using the split-marker system with primers Hmt10842up-F/HY and YG/Hmt10842down-R. A 500-bp probe (red bar) was amplified by polymerase chain reaction with Hmt6probe-F/Hmt6probe-R and used for Southern blot analysis. *Hin*dIII-digestion of genomic DNA from Br48 and Δmokset6 is expected to generate 1784-bp and 3942-bp DNA fragments, respectively. (**B**) Southern blot analysis using the probe and *Hin*dIII-digested genomic DNA.
(PDF)

S8 Fig Western blotting analysis of histone modifications in KMT mutants. Total protein extracted from *M*. *oryzae* cells was subjected to 15% SDS polyacrylamide gel electrophoresis, and probed with antibodies against H3K14me2 (active motif #39350), H3K36me3 (active motif #61102), and H3K79me2 (active motif #39144), respectively.
(PDF)

S9 Fig Western blot analysis of histone modifications in genetic complementation strains of the Δmokmt1, Δmoset1, Δmokmt5, and Δmokmt6 mutants. Total protein extracted from *M*. *oryzae* cells (wild type [WT], deletion mutants [Del], and complemented strains [C]) was subjected to 15% SDS_polyacrylamide gel electrophoresis, and probed with antibodies against H3K9me3 (Active Motif #39161), H4K20me3 (Active Motif #39181) and H3K4me2 (Active Motif #39141), respectively.
(PDF)

S10 Fig Inoculation test of the Δmoset1 mutant on the super susceptible barley cultivar Nigrate. Infection assay was performed at 22°C. Five days after inoculation, symptoms on the inoculated plants were evaluated. WT, Br48; Δset1, Δmoset1; Set1C, complemented strain of Δmoset1.
(PDF)

S11 Fig Inoculation test of the Δmoset1 mutant on wheat. Infection assay was performed using the wheat cultivar Norin4 at 22°C. (A) Spore suspension at a concentration of 1–2 × 105 spores/ml was with (+) or without (-) 5 μM 1, 16-hexadecanediol (cutin monomer) or 5mM cAMP was dropped onto intact wheat leaf surface. Inoculated leaves were incubated under dark and humid conditions for 24 h, and moved to an incubator at 22°C. Five days after inoculation, symptoms on the inoculated plants were evaluated. (B) Inoculation assay was performed as described in (A). Spore suspension was dropped to leaves with (+W) or without (C) a wound created by breaching the cuticle with a needle. WT, wild-type (Br48).
(PDF)

S12 Fig Phenotypic characterization of Δmoset1 mutants complemented with N-terminal FLAG-tagged MoSET1. Vegetative growth was measured for 7 days on complete agar medium. Conidiation was measured by counting the number of conidia harvested 3 days after BLB induction by suspending them with 20 ml of sterile distilled water per plate. Appressorium formation was measured as the percentage ratio of appressorium-forming mycelium to germinating mycelium on hydrophobic surfaces after 24 h incubation at 25°C. All data were given as ratio relative to average values in the wild-type strain Br48. Black bars indicate the original Δmoset1 mutant (Δmoset1.36). Gray bars represent two complemented transformants (TF2 and TF3) with N-terminal FLAG-tagged MoSET1. All data are presented as means ± SD from three replicates. *, significant difference from the wild-type strain (*p*< 0.01, two-tailed t-test after angular transformation).
(PDF)

S13 Fig Distribution of H3K4me2 and H3K4me3 across the complete set of *M*. *oryzae* genes. Average read density (number of reads per million reads) across all genes at a position relative to the known or presumed transcription start site (TSS) was plotted in the graph. H3K4me2 and H3K4me3 distribution patterns were shown in red and green lines, respectively. Solid and dashed lines indicate data in mycelia and germination tubes, respectively.
(PDF)

S1 Table *Magnaporthe oryzae* strains used in this study. (PDF)

S2 Table Summary of Next Generation Sequencing (NGS) analyses. (DOCX)

S3 Table List of genes differentially enriched for H3K4me2/me3 between mycelia and germtubes. (XLSX)

S4 Table Over-represented GO categories in genes differentially enriched for H3K4me2/me3 between mycelia and germtubes. (XLSX)

S5 Table List of genes differentially expressed between mycelia and germtubes in a *Moset1*-dependent or independent manner. (XLSX)

S6 Table Over-represented GO categories in genes differentially expressed between mycelia and germtubes in a *Moset1*-dependent or independent manner. (XLSX)

S7 Table Primers used in this study. (PDF)

## References

[1] Strathl BD, Allis CD (2000) The language of covalent histone modifications. Nature 403: 41–45. 10638745 10.1038/47412

[2] Shilatifard A (2008) Molecular implementation and physiological roles for histone H3 lysine 4 (H3K4) methylation. Curr Opin Cell Biol 20: 341–8. 10.1016/j.ceb.2008.03.019 18508253 PMC2504688

[3] Lachner M, Jenuwein T (2002) The many faces of histone lysine methylation. Curr Opin Cell Biol 14: 286–98. 12067650 10.1016/s0955-0674(02)00335-6

[4] Copeland RA, Solomon ME, Richon VM (2009) Protein methyltransferases as a target class for drug discovery. Nat Rev Drug Discov 8: 724–732. 10.1038/nrd2974 19721445

[5] Bedford MT, Richard S (2005) Arginine methylation an emerging regulator of protein function. Mol Cell 18: 263–272. 15866169 10.1016/j.molcel.2005.04.003

[6] Allis CD, Berger SL, Cote J, Dent S, Jenuwien T, Kouzarides T, Pillus L, Reinberg D, Shi Y, Shiekhattar R, Shilatifard A, Workman J, Zhang Y (2007) New nomenclature for chromatin-modifying enzymes. Cell 131: 633–636. 18022353 10.1016/j.cell.2007.10.039

[7] Schotta G1, Ebert A, Krauss V, Fischer A, Hoffmann J, Rea S, Jenuwein T, Dorn R, Reuter G (2002) Central role of Drosophila SU(VAR)3-9 in histone H3-K9 methylation and heterochromatic gene silencing. EMBO J. 21:1121–1131. 11867540 10.1093/emboj/21.5.1121 PMC125909

[8] Nakayama J, Rice JC, Strahl BD, Allis CD, Grewal SI (2001) Role of histone H3 lysine 9 methylation in epigenetic control of heterochromatin assembly. Science 292:110–113. 11283354 10.1126/science.1060118

[9] Tamaru H, Selker EU (2001) A histone H3 methyltransferase controls DNA methylation in *Neurospora crassa* . Nature 414: 277–283. 11713521 10.1038/35104508

[10] Nislow C, Ray E, Pillus L (1997) SET1, a yeast member of the trithorax family, functions in transcriptional silencing and diverse cellular processes. Mol Biol Cell 8: 2421–2436. 9398665 10.1091/mbc.8.12.2421 PMC25717

[11] Mozer BA, Dawid IB (1989) Cloning and molecular characterization of the trithorax locus of *Drosophila melanogaster* . Proc Nat Acad Sci USA 86: 3738–3742. 2566995 10.1073/pnas.86.10.3738 PMC287215

[12] Mareike A, Kristian H (2010) Histone methyltransferases in cancer. Semin Cell Dev Biol 21: 209–220 10.1016/j.semcdb.2009.10.007 19892027

[13] Zhou Z, Rune T, Søren K, Anders LN (2010) The NSD3L histone methyltransferase regulates cell cycle and cell invasion in breast cancer cells. Biochem Biophys Res Commun 398: 565–570. 10.1016/j.bbrc.2010.06.119 20599755

[14] Tamaru H, Zhang X, McMillen D, Singh PB, Nakayama J, Grewal SI, Allis CD, Cheng X, Selker EU (2003) Trimethylated lysine 9 of histone H3 is a mark for DNA methylation in *Neurospora crassa* . Nat Genet 34:75–79. 12679815 10.1038/ng1143

[15] Zachary AL, Adhvaryu KK, Honda S, Shiver A, Selker UE (2010) Identification of DIM-7, a protein required to target the DIM-5 H3 methyltransferase to chromatin. Proc Nat Acad Sci USA 107: 8310–8315. 10.1073/pnas.1000328107 20404183 PMC2889519

[16] Keyur KA, Morris SA, Strahl BD, Selker EU (2005) Methylation of histone H3 Lysine 36 is required for normal development in *Neurospora crassa* . Eukaryot Cell 4: 1455–1464. 16087750 10.1128/EC.4.8.1455-1464.2005 PMC1214527

[17] Jamieson K1, Rountree MR, Lewis ZA, Stajich JE, Selker EU (2013) Regional control of histone H3 lysine 27 methylation in *Neurospora* . Proc Nat Acad Sci USA 110:6027–6032. 10.1073/pnas.1303750110 23530226 PMC3625340

[18] Connolly LR, Smith KM, Freitag M. (2013) The *Fusarium graminearum* histone H3 K27 methyltransferase KMT6 regulates development and expression of secondary metabolite gene clusters. PLoS Genet 9:e1003916 10.1371/journal.pgen.1003916 24204317 PMC3814326

[19] Chujo T, Scott B. (2014) Histone H3K9 and H3K27 methylation regulates fungal alkaloid biosynthesis in a fungal endophyte-plant symbiosis. Mol Microbiol 92:413–34 10.1111/mmi.12567 24571357

[20] Soyer JL, El Ghalid M, Glaser N, Ollivier B, Linglin J, Grandaubert J, Balesdent MH, Connolly LR, Freitag M, Rouxel T, Fudal I. (2014) Epigenetic control of effector gene expression in the plant pathogenic fungus *Leptosphaeria maculans* . PLoS Genet 10:e1004227 10.1371/journal.pgen.1004227 24603691 PMC3945186

[21] Bok JW, Chiang YM, Szewczyk E, Reyes-Dominguez Y, Davidson AD, Sanchez JF, Lo HC, Watanabe K, Strauss J, Oakley BR, Wang CC, Keller NP (2009) Chromatin-level regulation of biosynthetic gene clusters. Nat Chem Biol 5: 462–464. 10.1038/nchembio.177 19448638 PMC2891026

[22] Palmer JM, Bok JW, Lee S, Dagenais TR, Andes DR, Kontoyiannis DP, Keller NP (2013) Loss of CclA, required for histone 3 lysine 4 methylation, decreases growth but increases secondary metabolite production in *Aspergillus fumigatus* . Peer J 1: e4 10.7717/peerj.4 23638376 PMC3629006

[23] Briggs SD, Bryk M, Strahl BD, Cheung WL, Davie JK, Dent SY, Winston F, Allis CD (2001) Histone H3 lysine 4 methylation is mediated by *Set1* and required for cell growth and rDNA silencing in *Saccharomyces cerevisiae* . Genes Dev 15: 3286–3295. 11751634 10.1101/gad.940201 PMC312847

[24] Berretta J, Pinskaya M, Morillon A (2008) A cryptic unstable transcript mediates transcriptional trans-silencing of the *Ty1* retrotransposon in *S*. *cerevisiae* . Genes Dev 22: 615–626. 10.1101/gad.458008 18316478 PMC2259031

[25] Cheng J1, Blum R1, Bowman C1, Hu D, Shilatifard A, Shen S, Dynlacht BD (2014) A role for H3K4 monomethylation in gene repression and partitioning of chromatin readers. Mol Cell 2014 53:979–992. 10.1016/j.molcel.2014.02.032 PMC4031464 24656132

[26] Xu JR, Zhao X, Dean RA (2007) From genes to genomes; a new paradigm for studying fungal pathogenesis in *Magnaporthe oryzae* . Adv Genet 57: 175–218. 17352905 10.1016/S0065-2660(06)57005-1

[27] Wilson RA, Talbot NJ (2009) Under pressure: investigating the biology of plant infection by *Magnaporthe oryzae* . Nat Rev Microbiol 7: 185–195. 10.1038/nrmicro2032 19219052

[28] Gowda M, Venu RC, Raghupathy MB, Nobuta K, Li H, Wing R, Stahlberg E, Couglan S, Haudenschild CD, Dean R, Nahm BH, Meyers BC, Wang GL (2006) Deep and comparative analysis of the mycelium and appressorium transcriptomes of *Magnaporthe grisea* using MPSS, RL-SAGE, and oligo array methods. BMC Genomics 7: 310 17156450 10.1186/1471-2164-7-310 PMC1764740

[29] Mathioni SM, Belo A, Rizzo CJ, Dean RA, Donofrio NM (2011) Transcriptome profiling of the rice blast fungus during invasive plant infection and *in vitro* stress. BMC Genomics. 12:49 10.1186/1471-2164-12-49 21247492 PMC3037901

[30] Soanes DM, Chakrabarti A, Paszkiewicz KH, Dawe AL, Talbot NJ (2012) Genome-wide transcriptional profiling of appressorium development by the rice blast fungus *Magnaporthe oryzae* . PloS Pathogen 8: e1002514. 22346750 10.1371/journal.ppat.1002514 PMC3276559

[31] Vu VB, Pham TMK, Nakayashiki H (2013) Substrate-induced transcriptional activation of the MoCel7C cellulase gens is associated with methylation of histone H3 at lysine 4 in the rice blast fungus *Magnaporthe oryzae* . Appl Environ Microbiol 79: 6823–6832. 10.1128/AEM.02082-13 23995923 PMC3811509

[32] Fu J, Hettler E, Wickes BL (2005) Split marker transformation increases homologous integration frequency in *Cryptococcus neoformans* . Fungal Genet Biol 43: 200–212 10.1016/j.fgb.2005.09.007 16497523

[33] Hyon G, Nguyen TTN, Chuma I, Inoue Y, Asano H, Murata N, Kusaba M, Tosa Y (2012) Charaterization of interactions between barley and various host-specific subgroups of *Magnaporthe oryzae* and *M*. *grisea* . J Gen Plant Pathol 78: 237–246.

[34] Ngueyn QB, Kadotani N, Kasahara S, Tosa Y, Mayama S, Nakayashiki H (2008) Systemic functional analysis of calcium signaling protein in the genome of the rice blast fungus, *Magnaporthe oryzae*, using a high-throughput RNA silencing system. Mol Microbiol 68: 1348–1365. 10.1111/j.1365-2958.2008.06242.x 18433453

[35] Gilbert RD, Johnson AM, Dean RA (1996) Chemical signals responsible for appressoirum formation n the rice blast fungus *M*. *grisea* . Physiol Mol Plant Pathol 48: 335–346.

[36] Lee YH, Dean RA (1993) cAMP regulates infection structure formation in the plant pathogenic fungus *Magnaporthegrisea* . Plant Cell 5: 693–700. 12271080 10.1105/tpc.5.6.693 PMC160306

[37] Mitchell TK, Dean RA (2005) The cAMP-dependent protein kinase catalytic subunit is required for appressorium formation and pathogenesis by the rice blast pathogen *Magnaporthe oryzae* . Plant Cell 7: 1869–1878. 10.1105/tpc.7.11.1869 PMC161045 8535140

[38] Wang Z, Gerstein M, Snyder M (2009) RNA-Seq: a revolutionary tool for transcriptomics. Nat Rev Genet 10: 57–63. 10.1038/nrg2484 19015660 PMC2949280

[39] Pokholok DK, Harbison CT, Levine S, Cole M, Hannett NM, Lee TI, Bell GW, Walker K, Rolfe PA, Herbolsheimer E, Zeitlinger J, Lewitter F, Gifford DK, Young RA (2005) Genome-wide map of nucleosome acetylation and methylation in yeast. Cell 122: 517–527. 16122420 10.1016/j.cell.2005.06.026

[40] Barski A, Cuddapah S, Cui K, Roh TY, Schones DE, Wang Z, Wei G, Chepelev I, Zhao K (2007) High-resolution profiling of histone methylations in the human genome. Cell 129: 823–837. 17512414 10.1016/j.cell.2007.05.009

[41] Van DK, Ding Y, Malkaram S, Riethoven JJ, Liu R, Yang J, Laczko P, Chen H, Xia Y, Ladunga I, Avramova Z, Fromm M (2010) Dynamic changes in genome-wide histone H3 lysine 4 methylation patterns in response to dehydration stress in *Arabidopsis thaliana* . BMC Plant Biol 10: 238 10.1186/1471-2229-10-238 21050490 PMC3095321

[42] Gilbert MJ, Thornton CR, Wakley GE, Talbot NJ (2006) A P-type ATPase required for rice blast disease and induction of host resistance. Nature 440: 535–539. 16554820 10.1038/nature04567

[43] James TC, Elgin SC (1986) Identification of a nonhistone chromosomal protein associated with heterochromatin in *Drosophila melanogaster* and its gene. Mol Cell Biol 6: 3862–3872. 3099166 10.1128/mcb.6.11.3862-3872.1986 PMC367149

[44] Bannister AJ, Schneider R, Myers FA, Thorne AW, Crane-Robinson C, Kouzarides T (2005) Spatial distribution of di- and tri-methyl lysine 36 of histone H3 at active genes. J Biol Chem 280: 17732–17736. 15760899 10.1074/jbc.M500796200

[45] Guenther MG, Levine SS, Boyer LA, Jaenisch R, Young RA (2007) A chromatin landmark and transcription initiation at most promoters in human cells. Cell 130: 77–88. 17632057 10.1016/j.cell.2007.05.042 PMC3200295

[46] Gregory GD, Vakoc CR, Rozovskaia T, Zheng X, Patel S, Nakamura T, Canaani E, Blobel GA (2007) Mammalian ASH1L is a histone methyltransferase that occupies the transcribed region of active genes. Mol Cell Biol 27: 8466–8479. 17923682 10.1128/MCB.00993-07 PMC2169421

[47] Tanaka Y, Kawahashi K, Katagiri Z, Nakayama Y, Mahajan M, Kioussis D (2011) Dual function of histone H3 lysine 36 methyltransferase ASH1 in regulation of Hox gene expression. PLoS ONE 6: e28171 10.1371/journal.pone.0028171 22140534 PMC3225378

[48] Jeon J, Goh J, Yoo S, Chi MH, Choi J, Rho HS, Park J, Han SS, Kim BR, Park SY, Kim S, Lee YH (2008) A putative MAP kinase kinase kinase, MCK1, is required for cell wall integrity and pathogenicity of the rice blast fungus, *Magnaporthe oryzae* . Mol Plant Microbe Interact 21: 525–34. 10.1094/MPMI-21-5-0525 18393612

[49] Liu XH, Lu JP, Zhang L, Dong B, Min H, Lin FC (2007) Involvement of a *Magnaporthe grisea* serine/threonine kinase gene, MgATG1, in appressorium turgor and pathogenesis. Eukaryot Cell 6: 997–1005. 17416896 10.1128/EC.00011-07 PMC1951528

[50] Nguyen BQ, Itoh K, Vu VB, Nakayashiki H (2012) Simultaneous silencing of endo-β-1,4xylanase genes reveals their roles in the virulence of *Magnaporthe oryzae* . Mol Microbiol 81: 1008–1019. 10.1111/j.1365-2958.2011.07746.x 21696466

[51] Vu VB, Itoh K, Nguyen BQ, Tosa Y, Nakayashiki H (2012) Cellulose belonging to glycoside hydrolase family 6 ans 7 contribute to the virulence of *Magnaporthe oryzae* . Mol Plant Microbe Interact 25: 1135–1141. 10.1094/MPMI-02-12-0043-R 22852807

[52] Raduwan H, Isola AL, Belden WJ (2013) Methylation of histone H3 on lysine 4 by the lysine methyltransferase SET1 protein is needed for normal clock gene expression. J Biol Chem 288:8380–8390. 10.1074/jbc.M112.359935 23319591 PMC3605655

[53] Urashima AS, Hashimoto Y, Don LD, Kusaba M, Tosa Y, Nakayashiki H, Mayama S (1999) Molecular analysis of the wheat blast population in Brazil with a homolog of retrotransposon MGR583. Annu Phytopathol Soc Jpn 65: 429–436.

[54] Nakayashiki H, Kiyotomi K, Tosa Y, Mayama S (1999) Transposition of the retrotransposon MAGGY in heterologous species of filamentous fungi. Genetics 153: 693–703 10511549 10.1093/genetics/153.2.693 PMC1460772

[55] Morita Y1, Hyon GS, Hosogi N, Miyata N, Nakayashiki H, Muranaka Y, Inada N, Park P, Ikeda K (2013) Appressorium-localized NADPH oxidase B is essential for aggressiveness and pathogenicity in the host-specific, toxin-producing fungus *Alternaria alternate* Japanese pear pathotype. Mol Plant Pathol. 14:365–378. 10.1111/mpp.12013 23279187 PMC6638787

[56] Kadotani N, Nakayashiki H, Tosa Y, Mayama S (2003) RNA silencing in the phytopathogenic fungus *Magnaporthe oryzae* . Mol Plant Microbe Interact 16: 769–775. 12971600 10.1094/MPMI.2003.16.9.769

[57] Murakami J, Tosa Y, Kataoka T, Tomita R, Kawasaki J, Chuma I, et al (2000) Analysis of host species specificity of *Magnaporthe grisea* toward wheat using a genetic cross between isolates from wheat and foxtail milliet. Phytopathology 90: 1060–1067. 10.1094/PHYTO.2000.90.10.1060 18944467

[58] Zhang SW, Mayama S, Tosa Y (2008) Identification of two genes for resistance to *Triticum* isolates of *Maganporthe oryzae* in wheat. Genome 56: 216–221. 10.1139/G07-094 18356957

[59] Kim D, Pertea G, Trapnell C, Pimentel H, Kelley R, Salzberg SL (2011) TopHat2: Accurate alignment of transcriptomes in the presence of insertions, deletions and gene fusions. Genome Biology 14:R36. 10.1186/gb-2013-14-4-r36 PMC4053844 23618408

[60] Li H., Durbin R. (2009) Fast and accurate short read alignment with Burrows-Wheeler Transform. Bioinformatics 25:1754–1760. 10.1093/bioinformatics/btp324 19451168 PMC2705234

[61] Robinson JT, Thorvaldsdóttir H, Winckler W, Guttman M, Lander ES, Getz G, Mesirov JP (2011) Integrative Genomics Viewer. Nature Biotechnology 29:24–26. 10.1038/nbt.1754 21221095 PMC3346182

[62] Robinson MD, McCarthy DJ, Smyth GK (2010) edgeR: a Bioconductor package for differential expression analysis of digital gene expression data. Bioinformatics 26:139–140 10.1093/bioinformatics/btp616 19910308 PMC2796818

[63] R Core Team (2013) R: A language and environment for statistical computing. R Foundation for Statistical Computing, Vienna, Austria http://www.R-project.org/.

[64] Robinson MD, Oshlack A (2010) A scaling normalization method for differential expression analysis of RNA-seq data. Genome Biology 11:R25 10.1186/gb-2010-11-3-r25 20196867 PMC2864565

[65] Benjamini Y, Hochberg Y (1995) Controlling the false discovery rate: a practical and powerful approach to multiple testing. J. R. Stat. Soc. Ser. B 57: 289–300.

[66] Falcon S., Gentleman R. (2007) Using GOstats to test gene lists for GO term association. Bioinformatics 23:257–258. 17098774 10.1093/bioinformatics/btl567

