# Correction: MoSET1 (Histone H3K4 Methyltransferase in *Magnaporthe oryzae*) Regulates Global Gene Expression during Infection-Related Morphogenesis

**DOI:** 10.1371/journal.pgen.1005752

**Published:** 2015-12-16

**Authors:** Kieu Thi Minh Pham, Yoshihiro Inoue, Ba Van Vu, Hanh Hieu Nguyen, Toru Nakayashiki, Ken-ichi Ikeda, Hitoshi Nakayashiki

The gene list under the H3K4me2 column on the enriched_in_GT tab in S3 Table is incorrect. Please view the correct S3 Table below.

## Supporting Information

S3 Table List of genes differentially enriched for H3K4me2/me3 between mycelia and germtubes. (XLSX)
