# Supplementary figures and images for: MoSET1 (Histone H3K4 Methyltransferase in Magnaporthe oryzae) Regulates Global Gene Expression during Infection-Related Morphogenesis
Source: PLoS Genet. 2015 Jul 31;11(7):e1005385. doi: 10.1371/journal.pgen.1005385 (PMC4521839; doi:10.1371/journal.pgen.1005385)

Figure S1 Pham et al. PLoS Genet.

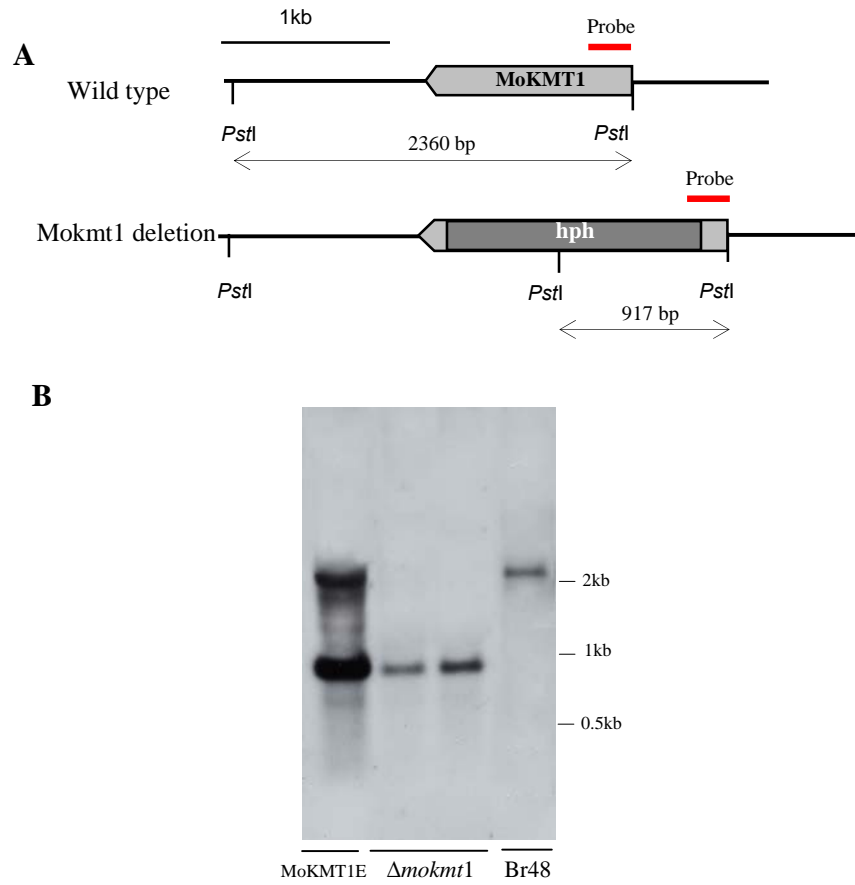

Supplement: S1 Fig — (A) Schematic representation of the Mokmt1 (MGG_06852) locus in the wild type (Br48) and Δmokmt1 strains. The map position of Mokmt1 is from 2,991,740 to 2,993,241 on chromosome supercont8.1 of Magnaporthe oryzae 70–15. Deletion strains were constructed using the split-marker system with primers Hmt18up-XhoI-F/HY and YG/Hmt18down-BlgII-R. A 214-bp probe (red bar) was amplified by polymerase chain reaction with Hmt18down-KpnI-F/Hmt18-sreR and used for Southern blot analysis. PstI-digestion of genomic DNA from Br48 and Δmokmt1 is expected to generate 2360-bp and 917-bp DNA fragments, respectively. (B) Southern blot analysis using the probe and PstI-digested genomic DNA. MoKMT1E indicates an ectopic mutant. (PDF) [file pgen.1005385.s001.pdf]

Figure S2 Pham et al. PLoS Genet.

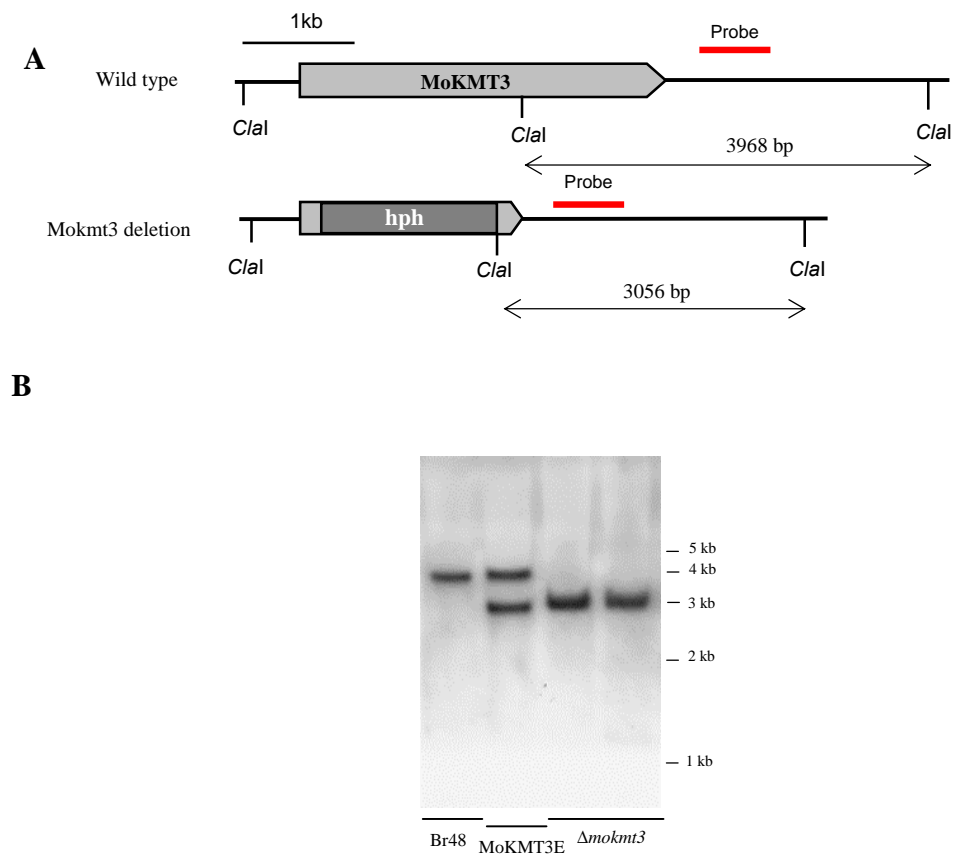

Supplement: S2 Fig — (A) Schematic representation of the Mokmt3 (MGG_01661) locus in the wild type (Br48) and Δmokmt3 strains. The map position of Mokmt3 is from 4,375,150 to 4,379,185 on chromosome supercont8.2 of Magnaporthe oryzae 70–15. Deletion strains were constructed using the split-marker system with primers 27up-F/HY and YG/Hmt27-1661down-BglII-R. A 429-bp probe (red bar) was amplified by polymerase chain reaction with Probe27-F/Probe27-R and used for Southern blot analysis. ClaI-digestion of genomic DNA from Br48 and Δ mokmt3 is expected to generate 3968-bp and 3056-bp DNA fragments, respectively. (B) Southern blot analysis using the probe and ClaI-digested genomic DNA. MoKMT3E indicates an ectopic mutant. (PDF) [file pgen.1005385.s002.pdf]

Figure S3 Pham et al. PLoS Genet.

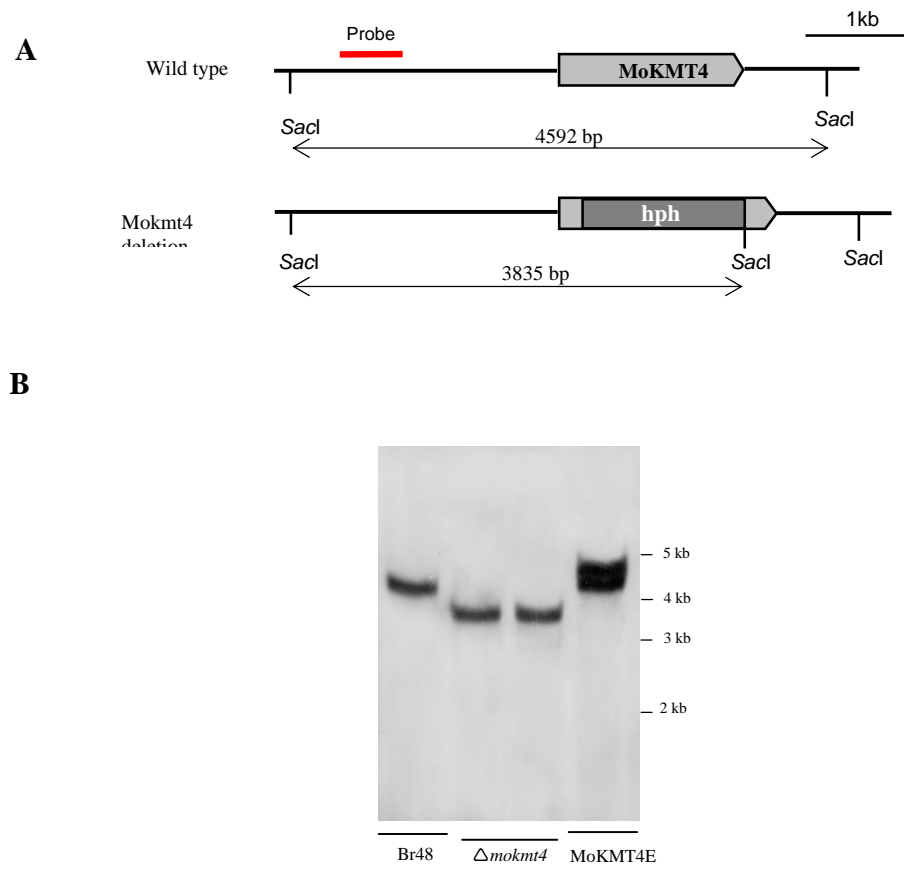

Supplement: S3 Fig — (A) Schematic representation of the Mokmt4 (MGG_05254) locus in the wild type (Br48) and Δmokmt4 strains. The map position of Mokmt4 is from 5,164,077 to 5,166,276 on chromosome supercont8.3 of Magnaporthe oryzae 70–15. Deletion strains were constructed using the split-marker system with primers Hmt22-05254-SphI-up-F/HY and YG/22down-R. A 491-bp probe (red bar) was amplified by polymerase chain reaction with H22probe-F/H22probe-R and used for Southern blot analysis. SacI-digestion of genomic DNA from Br48 and Δmokmt4 is expected to generate 4592-bp and 3835-bp DNA fragments, respectively. (B) Southern blot analysis using the probe and SacI-digested genomic DNA. MoKMT4E indicates an ectopic mutant. (PDF) [file pgen.1005385.s003.pdf]

Figure S4 Pham et al. PLoS Genet.

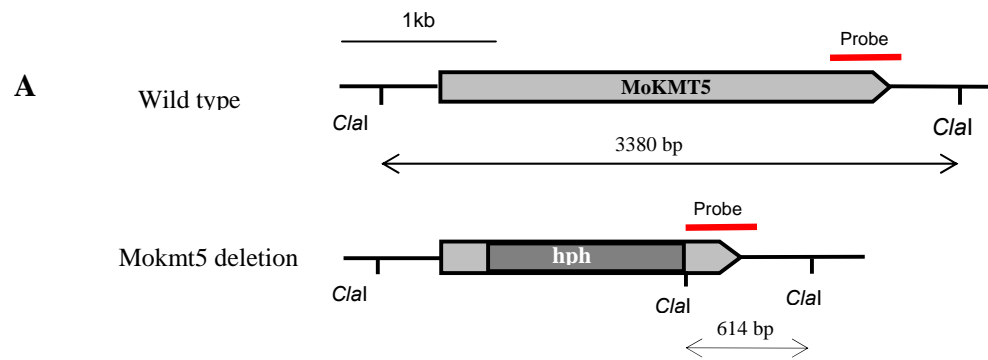

**B**

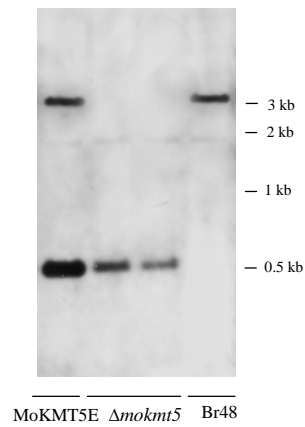

Supplement: S4 Fig — (A) Schematic representation of the Mokmt5 (MGG_07393) locus in the wild type (Br48) and Δmokmt5 strains. The map position of Mokmt5 is from 42,3851 to 427,601 on chromosome supercont8.3 of Magnaporthe oryzae 70–15. Deletion strains were constructed using the split-marker system with primers Set9up-F/HY and YG/Set9down-R. A 391-bp probe (red bar) was amplified by polymerase chain reaction with Set93down-F/Set9scree-R and used for Southern blot analysis. ClaI-digestion of genomic DNA from Br48 and Δmokmt5 strains is expected to generate 3380-bp and 614-bp DNA fragments, respectively. (B) Southern blot analysis using the probe and ClaI-digested genomic DNA. MoKMT5E indicates an ectopic mutant. (PDF) [file pgen.1005385.s004.pdf]

Figure S5 Pham et al. PLoS Genet.

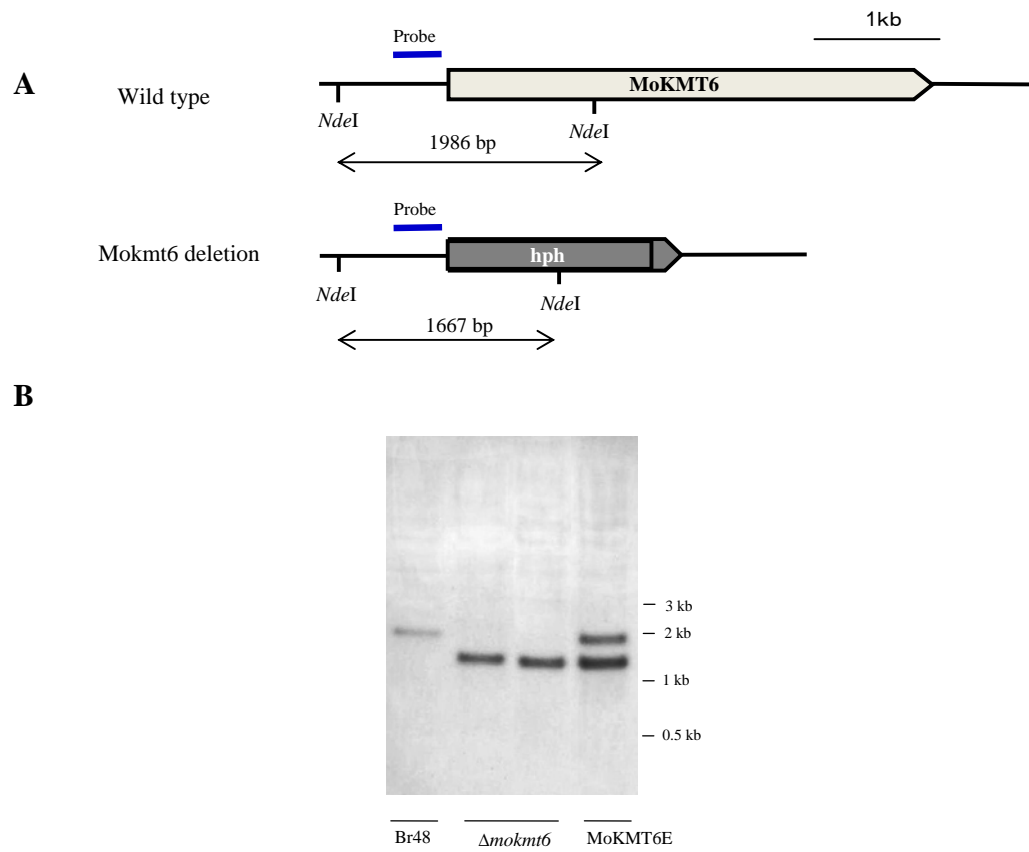

Supplement: S5 Fig — (A) Schematic representation of the Mokmt6 (MGG_00152) locus in the wild type (Br48) and Δmokmt6 strains. The map position of Mokmt6 is from 3,929,861–3,934,291 on chromosome supercont8.5 of Magnaporthe oryzae 70–15. Deletion strains were constructed using the split-marker system with primers Mgg-00152 up-F/HY and YG/Mgg-00152 down-R. A 463-bp probe (blue bar) was amplified by polymerase chain reaction with Mgg-00152probe-F/Mgg-00152probe-R and used for Southern blot analysis. NdeI-digestion of genomic DNA from Br48 and Δmokmt6 strains is expected to generate 1986-bp and 1667-bp DNA fragments, respectively. (B) Southern blot analysis using the probe and NdeI digested genomic DNA. MoKMT6E indicates an ectopic mutant. (PDF) [file pgen.1005385.s005.pdf]

Figure S6 Pham et al. PLoS Genet.

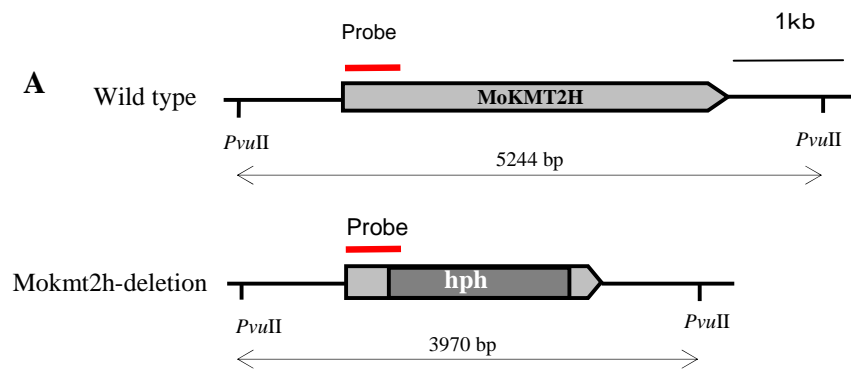

**B**

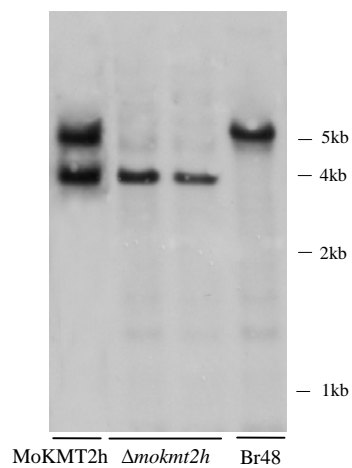

Supplement: S6 Fig — (A) Schematic representation of the Mokmt2h (MGG_02937) locus in the wild type (Br48) and Δmokmt2h strains. The map position of Mokmt2h is from 941,917 to 946,513 on chromosome supercont8.7 of Magnaporthe oryzae 70–15. Deletion strains were constructed using the split-marker system with primers 23up-HindIII-F/HY and YG/22down-BglII-R. A 508-bp probe (red bar) was amplified by polymerase chain reaction with Hmt23-srce-F/23up-SphI-R and used for Southern blot analysis. PvuII-digestion of genomic DNA from Br48 and Δmokmt2h strains is expected to generate 5244-bp and 3970-bp DNA fragments, respectively. (B) Southern blot analysis using the probe and PvuII-digested genomic DNA. (PDF) [file pgen.1005385.s006.pdf]

Figure S7 Pham et al. PLoS Genet.

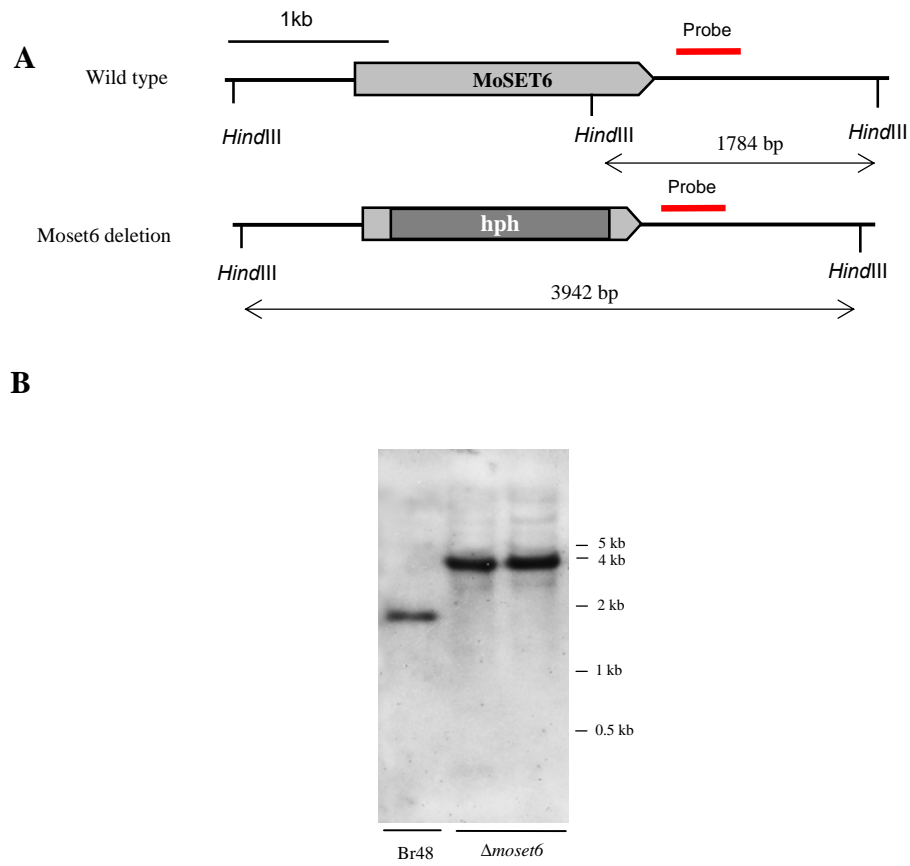

Supplement: S7 Fig — (A) Schematic representation of the Moset6 (MGG_15522) locus in the wild type (Br48) and Δmoset6 strains. The map position of Moset6 is from 494,343 to 496,716 on chromosome supercont8.2 of Magnaporthe oryzae 70–15. Deletion strains were constructed using the split-marker system with primers Hmt10842up-F/HY and YG/Hmt10842down-R. A 500-bp probe (red bar) was amplified by polymerase chain reaction with Hmt6probe-F/Hmt6probe-R and used for Southern blot analysis. HindIII-digestion of genomic DNA from Br48 and Δmokset6 is expected to generate 1784-bp and 3942-bp DNA fragments, respectively. (B) Southern blot analysis using the probe and HindIII-digested genomic DNA. (PDF) [file pgen.1005385.s007.pdf]

Figure S8 Pham et al. PLoS Genet.

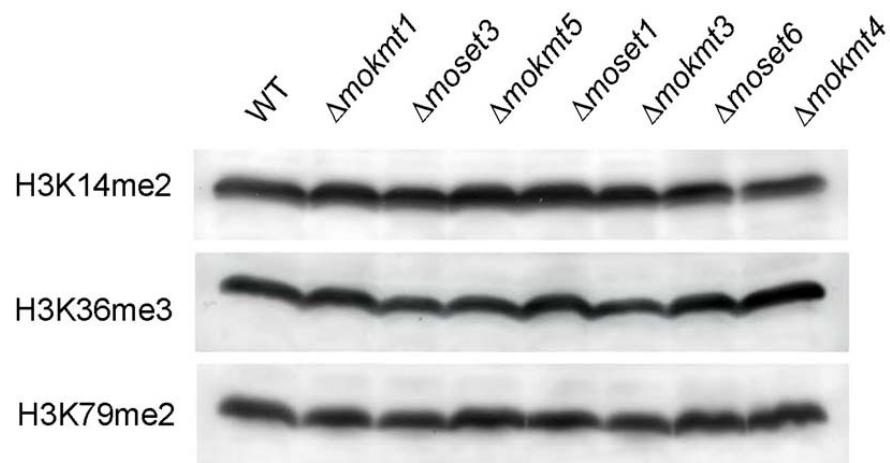

Supplement: S8 Fig — Total protein extracted from M. oryzae cells was subjected to 15% SDS polyacrylamide gel electrophoresis, and probed with antibodies against H3K14me2 (active motif #39350), H3K36me3 (active motif #61102), and H3K79me2 (active motif #39144), respectively. (PDF) [file pgen.1005385.s008.pdf]

Figure S9 Pham et al. PLoS Genet.

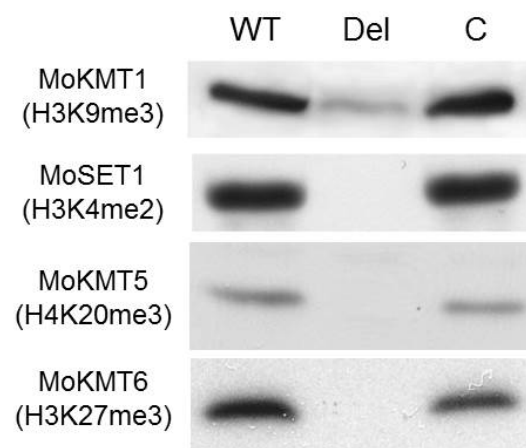

Supplement: S9 Fig — Total protein extracted from M. oryzae cells (wild type [WT], deletion mutants [Del], and complemented strains [C]) was subjected to 15% SDS_polyacrylamide gel electrophoresis, and probed with antibodies against H3K9me3 (Active Motif #39161), H4K20me3 (Active Motif #39181) and H3K4me2 (Active Motif #39141), respectively. (PDF) [file pgen.1005385.s009.pdf]

Figure S10 Pham et al. PLoS Genet.

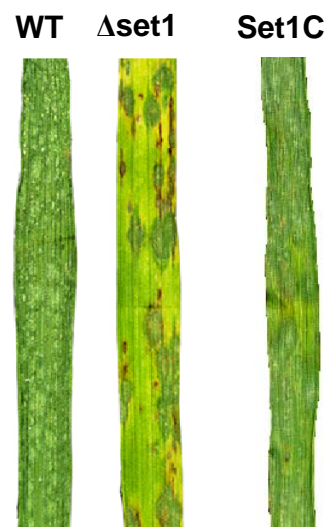

Supplement: S10 Fig — Infection assay was performed at 22°C. Five days after inoculation, symptoms on the inoculated plants were evaluated. WT, Br48; Δset1, Δmoset1; Set1C, complemented strain of Δmoset1. (PDF) [file pgen.1005385.s010.pdf]

Figure S11 Pham et al. PLoS Genet.

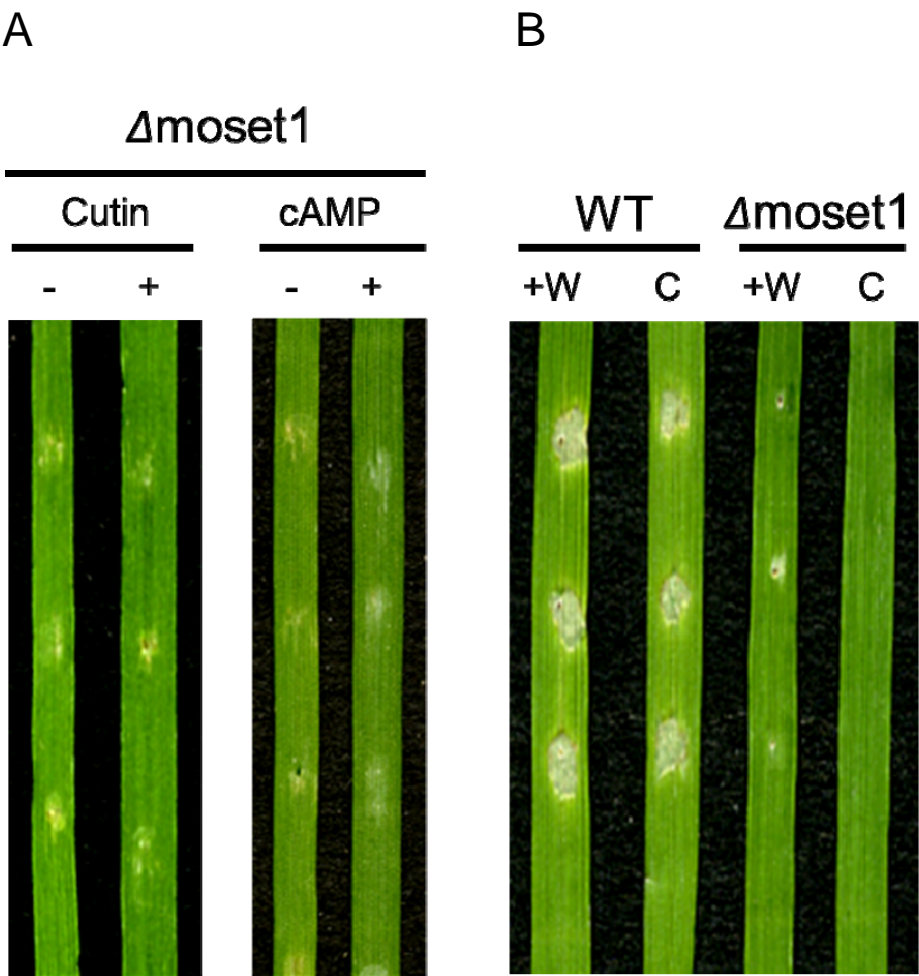

Supplement: S11 Fig — Infection assay was performed using the wheat cultivar Norin4 at 22°C. (A) Spore suspension at a concentration of 1–2 × 10^5^ spores/ml was with (+) or without (-) 5 μM 1, 16-hexadecanediol (cutin monomer) or 5mM cAMP was dropped onto intact wheat leaf surface. Inoculated leaves were incubated under dark and humid conditions for 24 h, and moved to an incubator at 22°C. Five days after inoculation, symptoms on the inoculated plants were evaluated. (B) Inoculation assay was performed as described in (A). Spore suspension was dropped to leaves with (+W) or without (C) a wound created by breaching the cuticle with a needle. WT, wild-type (Br48). (PDF) [file pgen.1005385.s011.pdf]

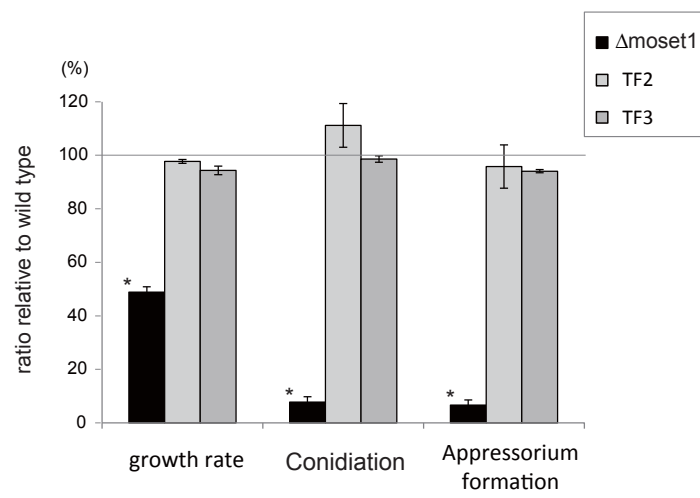

Supplement: S12 Fig — Vegetative growth was measured for 7 days on complete agar medium. Conidiation was measured by counting the number of conidia harvested 3 days after BLB induction by suspending them with 20 ml of sterile distilled water per plate. Appressorium formation was measured as the percentage ratio of appressorium-forming mycelium to germinating mycelium on hydrophobic surfaces after 24 h incubation at 25°C. All data were given as ratio relative to average values in the wild-type strain Br48. Black bars indicate the original Δmoset1 mutant (Δmoset1.36). Gray bars represent two complemented transformants (TF2 and TF3) with N-terminal FLAG-tagged MoSET1. All data are presented as means ± SD from three replicates. *, significant difference from the wild-type strain (p< 0.01, two-tailed t-test after angular transformation). (PDF) [file pgen.1005385.s012.pdf]

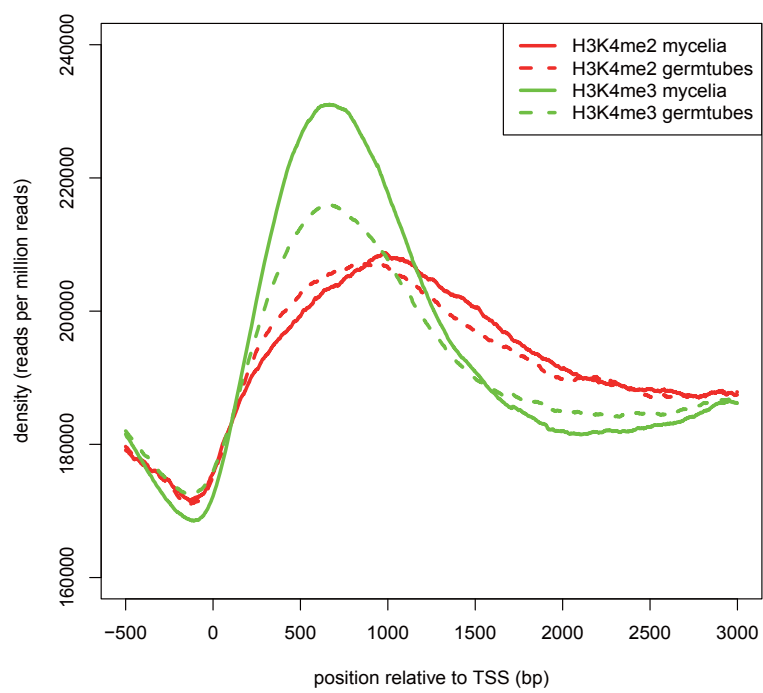

Supplement: S13 Fig — Average read density (number of reads per million reads) across all genes at a position relative to the known or presumed transcription start site (TSS) was plotted in the graph. H3K4me2 and H3K4me3 distribution patterns were shown in red and green lines, respectively. Solid and dashed lines indicate data in mycelia and germination tubes, respectively. (PDF) [file pgen.1005385.s013.pdf]
