# Supplementary material for: MoSET1 (Histone H3K4 Methyltransferase in Magnaporthe oryzae) Regulates Global Gene Expression during Infection-Related Morphogenesis
Source: PLoS Genet. 2015 Jul 31;11(7):e1005385. doi: 10.1371/journal.pgen.1005385 (PMC4521839; doi:10.1371/journal.pgen.1005385)
Supplement: S1 Table — (PDF) [file pgen.1005385.s014.pdf]

**Table S1. *Magnaporthe oryzae* strains used in this study**

| Strain | Genotype | Reference |
|---|---|---|
| Br48 | Wild type (wheat infecting isolate) | Urashima *et al.*, 1999 |
| Δ*mokmt1*.9 | *Mokmt1* deletion mutant of Br48 | This study |
| Δ*mokmt1*.13* | *Mokmt1* deletion mutant of Br48 | This study |
| MoKMT1E | Ectopic transformant for Δ*mokmt1* deletion mutant | This study |
| MoKMT1C | Complementation strain of Δ*mokmt1*.9 mutant | This study |
| Δ*moset1*.35 | *Moset1* deletion mutant of Br48 | Vu *et al.* 2013 |
| Δ*moset1*.36* | *Moset1* deletion mutant of Br48 | Vu *et al.* 2013 |
| MoSET1.19E | Ectopic transformant for Δ*moset1* deletion mutant | Vu *et al.* 2013 |
| MoSET1C | Complementation strain of Δ*moset1*.36 mutant | Vu *et al.* 2013 |
| Δ*mokmt3*.22* | *Mokmt3* deletion mutant of Br48 | This study |
| Δ*mokmt3*.26 | *Mokmt3* deletion mutant of Br48 | This study |
| MoKMT3E | Ectopic transformant for Δ*mokmt3* deletion mutant | This study |
| MoKMT3C | Complementation strain of Δ*mokmt3*.26 mutant | This study |
| Δ*mokmt4*.17* | *Mokmt4* deletion mutant of Br48 | This study |
| Δ*mokmt4*.19 | *Mokmt4* deletion mutant of Br48 | This study |
| MoKMT4E | Ectopic transformant for Δ*mokmt4* deletion mutant | This study |
| Δ*mokmt5*.2* | *Mokmt5* deletion mutant of Br48 | This study |
| Δ*mokmt5*.4 | *Mokmt5* deletion mutant of Br48 | This study |
| MoKMT5E | Ectopic transformant for Δ*mokmt5* deletion mutant | This study |
| MoKMT5C | Complementation strain of Δ*mokmt5*.2 mutant | This study |
| Δ*mokmt6*.5* | *Mokmt6* deletion mutant of Br48 | This study |
| Δ*mokmt6*.10 | *Mokmt6* deletion mutant of Br48 | This study |
| MoKMT6E | Ectopic transformant for Δ*mokmt6* deletion mutant | This study |
| MoKMT6C | Complementation strain of Δ*mokmt6*.5 mutant | This study |
| Δ*mokmt2h*.8 | *Mokmt2h* deletion mutant of Br48 | This study |
| Δ*mokmt2h*.13* | *Mokmt2h* deletion mutant of Br48 | This study |
| MoKMT2hE | Ectopic transformant for Δ*mokmt2h* deletion mutant | This study |
| MoKMT2hC | Complementation strain of Δ*mokmt2h*.13 mutant | This study |
| Δ*moset6*.14* | *Moset6* deletion mutant of Br48 | This study |
| Δ*moset6*.16 | *Moset6* deletion mutant of Br48 | This study |
| MoSET6E | Ectopic transformant for Δ*moset6* deletion mutant | This study |

*representative strains were used throughout this study unless otherwise noted.
