# Supplementary material for: MoSET1 (Histone H3K4 Methyltransferase in Magnaporthe oryzae) Regulates Global Gene Expression during Infection-Related Morphogenesis
Source: PLoS Genet. 2015 Jul 31;11(7):e1005385. doi: 10.1371/journal.pgen.1005385 (PMC4521839; doi:10.1371/journal.pgen.1005385)
Supplement: S2 Table — (DOCX) [file pgen.1005385.s015.docx]

**Table S2. Summary of Next Generation Sequencing (NGS) Analyses.**

| **^*1^ NGS_ID** | **^*2^ Strain** | **^*3^ Stage** | **^*4^Antibody** | **Accession** | **Parsed reads** | **Mapped reads** | **^*5^ %** |
| --- | --- | --- | --- | --- | --- | --- | --- |
| **a. RNA-seq** | |  |  |  |  |  |  |
| RS4WM | WT | myc | N/A | DRX019597 | 16,631,292 | 7,581,678 | 45.59 |
| RS5WM | WT | myc | N/A | DRX019601 | 12,120,620 | 5,638,237 | 46.52 |
| RS4WG | WT | GT | N/A | DRX019596 | 15,432,828 | 5,479,815 | 35.51 |
| RS5WG | WT | GT | N/A | DRX019600 | 9,791,812 | 3,549,629 | 36.25 |
| RS2SM | Δmoset1 | myc | N/A | DRX019593 | 4,995,254 | 2,141,518 | 42.87 |
| RS4SM | Δmoset1 | myc | N/A | DRX019595 | 15,352,676 | 6,586,377 | 42.90 |
| RS5SM | Δmoset1 | myc | N/A | DRX019599 | 13,423,494 | 5,781,722 | 43.07 |
| RS4SG | Δmoset1 | GT | N/A | DRX019594 | 22,067,774 | 8,275,442 | 37.50 |
| RS5SG | Δmoset1 | GT | N/A | DRX019598 | 9,784,324 | 3,972,780 | 40.60 |
| **b. ChIP-seq** | |  |  |  |  |  |  |
| CS2WM | WT | myc | H3K4me2 | DRX019590 | 5,5417,88 | 5,160,056 | 93.11 |
| CS2WG | WT | GT | H3K4me2 | DRX019589 | 5,145,166 | 4,946,169 | 96.13 |
| CS2WG-r | WT | GT | H3K4me2 | DRX030399 | 7,281,384 | 7,009,233 | 96.26 |
| CS3WM | WT | myc | H3K4me3 | DRX019592 | 8,467,364 | 8,261,375 | 97.57 |
| CS3WM-r | WT | myc | H3K4me3 | DRX030401 | 8,499,116 | 8,299,671 | 97.65 |
| CS3WG | WT | GT | H3K4me3 | DRX019591 | 7,779,596 | 7,360,835 | 94.62 |
| CS3WG-r | WT | GT | H3K4me3 | DRX030400 | 12,249,390 | 11,589,122 | 94.61 |
| CS0WM | WT | myc | H3 | DRX019588 | 4,048,838 | 3,689,903 | 91.13 |
| CS0WM-r | WT | myc | H3 | DRX030398 | 5,518,302 | 5,042,660 | 91.50 |
| CSsetM2 | Δmoset1-TF2 | myc | FLAG | DRX029805 | 2,127,736 | 1,378,441 | 64.88 |
| CSsetM3 | Δmoset1-TF3 | myc | FLAG | DRX029807 | 1,310,706 | 619,107 | 47.31 |
| CSsetG2 | Δmoset1-TF2 | GT | FLAG | DRX029804 | 3,435,736 | 3,066,607 | 89.33 |
| CSsetG3 | Δmoset1-TF3 | GT | FLAG | DRX029806 | 2,804,872 | 2,398,974 | 85.57 |

Samples were subjected to 75-nt or 120-nt pair-end sequencing.

*1**,** “-r” in NGS_ID indicates a technical replicate.

*2, WT, wild-type Br48 strain; Δmoset1-TF2 and -TF3,Δmoset1 transformants with FLAG-tagged MoSET1

*3, myc, vegetative mycelia; GT, germination tubes

*4, Antibodies used were listed in Materials and Methods

*5, relatively low percentages of mapped reads in RNA-seq analyses were due to the presence of cDNA derived from mitochondorial RNA species that were not mapped to the genome sequence we used.
