# Supplementary material for: MoSET1 (Histone H3K4 Methyltransferase in Magnaporthe oryzae) Regulates Global Gene Expression during Infection-Related Morphogenesis
Source: PLoS Genet. 2015 Jul 31;11(7):e1005385. doi: 10.1371/journal.pgen.1005385 (PMC4521839; doi:10.1371/journal.pgen.1005385)
Supplement: S7 Table — (PDF) [file pgen.1005385.s020.pdf]

Table S7. Primers used in this study

Primer	Sequence
Hmt18up-XhoI-F	5'-CTCGAGGTGCAAGGTTGAGCATCTGA-3'
Hmt18up-SalI-R	5'-GTCGACCCCTCTCCGAGAATCCTTTC-3'
Hmt18down-KpnI-F	5'-GGTACCCCCGTTTCTTCAACCATAGC-3'
Hmt18down-BglII-R	5'-AGATCTATCGCCAGAGAGTGGTGAAC-3'
Hmt18-sre F	5-ACATGGAGGGCAGGATGTAG-3'
Hmt18-sre R	5'-CCCCTGCAGTTAGTGCTTTT-3'
18-Full-F	5'-TTGAGCACTGAGATCCAACG-3'
18-Full-R	5'-CTGGCACACGGAATTATCCT-3'
Hmt23up-HindIII-F	5'-ACGAAGCTTCATCGAGAACTCAAGCGTCA-3'
Hmt23up-SphI-R	5'-TAGGCATGCTGGTACGTCTCCTCCTGTCC-3'
Hmt23down-KpnI-F	5'-CTAGGTACCAGACCAAGCAAGGGAAAACA-3'
Hmt23down-BglII	5'-GGCAGATCTGGCAGCAGGAAGTACCAGAC-3'
Hmt23-srce-F	5'-TACATCCAGACGCGTCATTC-3'
Hmt23-Scre-R	5'-CCATTTGCCCAGATACTCGT-3'
Hmt23comple-F	5'-CTGTCGCGTTTGTGACACTC-3'
Hmt23comple-R	5'-ACACCGCAAACCTGAAATGT-3'
Set9up-F	5'-CGGTGCTCTGTCTCTAGTTTCA-3'
Set9up-R	5'-GCGCATCTGTCAGTATGTCATC-3'
Set9down-F	5'-CTAAGGGCAAGATCGACGAG-3'
Set 9down-R	5'-CGAACTGAATTCGAAATCGGAACG-3'
Set9scree-F	5'-CCGACTGTACGACTCAGATGG-3'
Set9scree-R	5'-ACCCATCACGTCTTCACCTC-3'
Hmt3full F	5'-ATCCCTTCGTCGCTAGTTTG-3'
Hmt3full R	5'-ATATCACTCTGATACGAAACCCATC-3'
29up-F	5'-GCCGCAACCTCTACCAATGC-3'
29up-R	5'-TCCGGTATCCAACCTAGTGC-3'
29down-F	5'-CCCTGCCTGTGTGGAACTAC-3'
29down-R	5'-GTCCGTTCTCGGTGGTCTCT-3'
Scree29- F	5'-TGAGAGACATTGCACGGAGT-3'
Scree29- R	5'-GTCATCAAAGCCGAATCGAG-3'
29 full-F	5'-CAATTACCTCTTAACAACGACACC-3'
29 full-R	5'-TGACATGCGACTTCGGACT-3'
27up-F	5'-GCAATCTGTTTGCCTATGAAAT-3'
27up-R	5'-TGGGTCTCCATCTTGACGA-3'
Hmt27-1661down BamHI-F	5'-GGATCCGAACTGGACGATGCCGTTAT-3'
Hmt27-1661down BglI-R	5'-AGATCTCCCAATCGGATACAACAACC-3'
27screening-F	5'-CGCGTCTGATTATGGACGAC-3'
27screening-R	5'-GATGCAGTCCGAGTCTTCG-3'
Probe27-F	5'-GGGACGAGAACTGCTGAAAG-3'
Probe27-R	5'-TTGTAGACGAGGTGCAGACG-3'
27full-F	5'-AAGCGTACCCTCCCTTTGTC-3'
27full-R	5'-GGTGAAAGTATGTCAAAACTCAAGC-3'
Hmt10842up-F	5'-GTGGCAAGCGAAGACTCATT-3'
Hmt10842up-R	5'-AGTGGTGACATGTCGTCGAG-3'
Hmt610842down-F	5'-TGAGGAGGTGGGTGAAGAAC-3'
Hmt10842down-R	5'-GCAGGCTTGGTTTGACTAGC-3'
Scree10842-F	5'-ATGACTGGGAGGTCTTGCAC-3'
Scree10842-R	5'-CCTCGATGCATACGAATCCT-3'
Hmt6probe-F	5'-GGTACCTACCTGGTTACGGTGAG-3'
Hmt6probe-R	5'-CCTGAGTGGTTCAATACCACCTAC-3'
Hmt22-05254up-SphI-F	5'-GCATGCTACCCCTTTCCTACCGCTTT-3'
Hmt22-05254up-SalI-R	5'-GCATGCGCTTGCGCTGATACTGTTGT-3'
Hmt22-05254down-BamHI-F	5'-CGACCTAGTCCGAATATTCCTC-3'
22down-R	5'-CTACTATCTATCTCACCGCCAGCA-3'
H22 probe-F	5'-GCGGTTACTCACCGTAAAGC-3'
H22 probe-R	5'-AAGCAGGCAACCAACCATAC-3'
Mgg_07868 RT2-F	5'-CAGCAGAAGCAGGTGTACAAGA-3'
Mgg_07868 RT2-R	5'-AGCTGAAGGGGTCGTACAGAT-3'
Mgg_06164 RT 100 F	5'-TTTTCCCACGATGCCTACCT-3'
Mgg_06164 RT 100 R	5'-AAAGTATACGTTGCCCTCGAC-3'
Mgg_05912 RT F	5'-GTCGAGGGGCTGTTGGAC-3'
Mgg_05912 RT R	5'-TCTTCTCCTCAACCTCGCAA-3'
Mgg_00152 up-F	5'-TAAGTCCGGTAGTGTGAAGAGG-3'
Mgg_00152 up-R	5'-CTTGTCACTTTTGCTCCCTCC-3'
Mgg_00152 down-F	5'-ATCGGTGGAAGATGAGGACGAT-3'
Mgg_00152 down-R	5'-GTTGCTGGAGAGTACGTAACAA-3'
Mgg-00152 probe-F	5'-GCCCCATTTCCAACCCTAAC-3'

Mgg-00152 probe-R	5’-CACTCATGGCCTTCGGAAAC-3’
Mgg-00152 comple-R	CAATGGGTACTTTAGGCGGC
HY	5’-GGCTGCAGAACAGCGGGCAGTTCGG-3’
YG	5’-GCCGTGCACAGGGTGTCACGTTGC-3’
FLAG-MoSET1-F	5’ATGGACTACAAAGACGATGACGACAAGGACTACAAAGACGATGACGACAAGCCT CCGCCGCCCTCGAGCTTCACCGCATG-3’
MoSET1-TGA-R	5’-TCAGTTGAGGAAGCCCTTGCAGTTGGTAG-3’
IF-PMoSET-F	5’-CTGCAGGAATTCGATTTGGACAACCAGTTT-3’
IF-PMoSET-R	5’-GTCTTTGTAGTCCATTATATTATTGACACG-3’
IF-TMoSET-F	5’-GGCTTCCTCAACTGAAGTCATCTGCATTTT-3’
IF-TMoSET-R	5’-ATCGATAAGCTTGATTCCTTCACACTGGGA-3’
